# Hydrochemistry and environmental isotope signatures (δ^18^O and δ^2^H) in the crystalline basement groundwater system of Oye-Ekiti, southwestern Nigeria

**DOI:** 10.1038/s41598-026-50252-4

**Published:** 2026-04-27

**Authors:** Taiwo A. Bolaji, Zelalem K. Bedaso, Panagiotis G. Papazotos, Charles A. Oyelami, Jude R. Ogudo, Abel O. Olowoniyi

**Affiliations:** 1 Department of Geology, Federal University Oye-Ekiti, Oye-Ekiti, 371010 Nigeria; 2 Department of Earth and Environmental Geosciences, University of Dayton, Dayton, OH USA; 3 Department of Geological Sciences, School of Mining and Metallurgy Engineering, National Technical University of Athens, Athens, Greece; 4 Hellenic Survey of Geology and Mineral Exploration, 1 Sp. Louis Str., 13677 Acharnae, Greece

**Keywords:** Water quality, Geochemical modeling, Sustainable development goals, Stable isotopes, Multivariate statistics, Oye-Ekiti, Biogeochemistry, Chemistry, Environmental sciences, Hydrology, Solid Earth sciences

## Abstract

Groundwater is the primary source of domestic water supply in Oye-Ekiti, a rapidly urbanizing city in southwestern Nigeria. However, the processes controlling its quality and recharge remain poorly constrained. This study presents the first city-scale, dry-season assessment integrating hydrochemistry, stable isotopes (δ^18^O and δ^2^H), multivariate statistics, and geochemical modelling. Fifty samples collected from boreholes and hand-dug wells were analysed for physicochemical parameters, major ions, and environmental isotopes. Groundwater exhibits Ca–Mg–HCO_3_ and Na–K–Cl–SO_4_ as the primary water types, under acidic to slightly alkaline pH conditions, while total dissolved solids are generally below 500 mg/L. Ionic relationships and multivariate analyses identify silicate weathering and cation-exchange processes as the principal controls on groundwater chemistry, with localized nitrate and phosphate enrichment linked to agricultural activities and on-site sanitation. PHREEQC modelling revealed that all mineral phases, including calcite, dolomite, gypsum, and halite, are undersaturated, confirming active rock-water interaction. Stable isotope compositions (δ^18^O: − 3.53‰ to − 2.68‰; δ^2^H: − 17.65‰ to − 11.96‰) and the δ^18^O–δ^2^H groundwater regression line plots close to the regional Local Meteoric Water Line (LMWL) [δ^2^H = 7.7 × δ^18^O + 10.2, r^2^ = 0.87], indicating that the groundwater is predominantly of meteoric origin and derived from recent precipitation. The d-excess values (8.7–12.2‰), clustering around the global average of 10‰, suggest limited evaporative influence prior to recharge. Slight isotopic enrichment and higher solute variability in shallow hand-dug wells highlight their greater vulnerability to surface processes. These findings provide a new geochemical and isotopic baseline, providing critical insights for sustainable groundwater management, monitoring, and policy implementation under Sustainable Development Goals (SDG) 6 in a rapidly urbanizing environment.

**Supplementary Information:**

The online version contains supplementary material available at 10.1038/s41598-026-50252-4.

## Introduction

Groundwater resources are increasingly becoming the main source of potable water in major cities in Nigeria^1–3^ for many reasons, which include inadequate supply of pipe-borne water by the government^4^, growing population of the country^5^, vulnerability of surface waters to contamination^6^, and other economic factors. However, poor management of groundwater resources due to anthropogenic activities poses a threat to this valuable resource^7,8^. Although groundwater quality varies within the region, especially in relation to lithology and anthropogenic activities, the availability, movement, and chemistry of groundwater in Oye-Ekiti and its environs are mainly controlled by secondary porosity and permeability in the host rocks^9,10^. These secondary features give rise to two hydraulically connected aquifer systems: (i) the shallow weathered regolith and (ii) the deeper fractured basement aquifer. Areas with higher fracture density and thicker regolith zones typically facilitate rapid recharge and short groundwater residence times.

Oye-Ekiti is a rapidly-growing city in southwestern Nigeria. More specifically, as a town that hosts the vast student population, attention must be given to building a sustainable city and community in line with Goal 11 of the United Nations’ Sustainable Development Goals (SDGs). Sustainable cities are built around clean water and adequate sanitation (SDG 6) as a prerequisite for good health and well-being (SDG 3) UNSDG^97^^11^,. The rapid population growth, urban expansion, and increased dependence on shallow hand-dug wells have raised concerns about groundwater vulnerability. This vulnerability stems from geogenic inputs, evaporative concentration during the dry season, and contamination from sanitation systems and agricultural activities. Despite this growing reliance on groundwater, the hydrogeochemical processes that govern groundwater quality in the Oye-Ekiti crystalline basement aquifer are still not well understood.

External factors, including climate change and anthropogenic activities significantly affect the hydrochemical characteristics and development of groundwater systems^12–14^. Additionally, the origin of aquifer materials, the prevailing hydrogeological conditions, and inputs from run-off recharge determine groundwater availability, spatial distribution, and quality within a catchment area^15–17^.

In monitoring and quality assessment of groundwater, stable isotopes are used to analyze the sources of dissolved ions and the hydrogeochemical processes that control the aquifer system^18–20^. Tamez-Melendez et al.^28^. Similarly, studies have been employed to provide valuable information on the origin and quality of groundwater in the basement complex of southwestern Nigeria^10,21^.

The isotopic signature of a few chosen anthropogenic sulfate sources in Poland’s most urbanized and industrialized area was examined by Jakobczyk-Karpierz and Slosarczyk^94^; the findings were contrasted with published data from other regions of the world^22–25^ Pradhan et al. (2022). The study recommended combining chemical investigations with stable-isotope analyses to provide a comprehensive evaluation of contaminants’ fate and the origin of groundwater in metropolitan areas. Stable isotopes of oxygen (δ^18^O) and hydrogen (δ^2^H) serve as conservative dual tracers that provide valuable insights into water movement within the hydrologic cycle^26^ and the processes governing groundwater recharge^27^. These methods have also been applied in various regions. For example, Alemayehu et al.^19^ employed stable isotope data (δ^2^H, δ^18^O, and δ^13^C) in combination with hydrogeochemical analyses to investigate groundwater chemistry in the central part of the Mekelle sedimentary terrain, demonstrating the effectiveness of these tools in characterizing aquifer properties and tracing groundwater chemical evolution. Similarly, Tamez-Meléndez et al.^28^ integrated environmental isotopes with groundwater chemistry to elucidate the occurrence and dynamics of groundwater in the coastal aquifers of La Paz, California.

These isotopic approaches have been effectively applied in various regions of Nigeria. Ohwoghere-Asuma et al.^29^ established a Local Meteoric Water Line (LMWL, δ^2^H = 7.7 × δ^18^O + 10.2) using precipitation samples from three sites in the Niger Delta, providing a baseline for water resource assessments. Similarly, based on the Cotonou (Republic of Benin) Global Network of Isotopes in Precipitation (GNIP) data, Yusuf et al.^30^ reported an identical LMWL (δ^2^H = 7.7 × δ^18^O + 9.1). In southwestern Nigeria, Aladejana et al.^31^ utilized stable isotope techniques to characterize groundwater origin, recharge mechanisms, and residence time. Talabi and Tijani^10^ also investigated the basement aquifer system in Ekiti and demonstrated that the local shallow groundwater is primarily recharged by precipitation. Although these studies have applied hydrochemistry and stable isotope analyses to basement aquifers in southwestern Nigeria^10,30,31^, significant knowledge gaps remain for Oye-Ekiti. Firstly, no city-scale hydrogeochemical baseline exists to describe dominant ion sources, rock-water interactions, or the degree of anthropogenic influence. In addition, stable isotope (δ^2^H–δ^18^O) data are completely absent for the area, leaving recharge sources and evaporative conditions unknown. Furthermore, the specific differences in groundwater chemistry between shallow hand-dug wells and deeper boreholes have never been systematically evaluated. Also, most regional studies were conducted under wet-season or mixed-season conditions; the dry-season behaviour, when water tables are lowest and evaporative enrichment can be highest, remains unexplored. Finally, no previous study has integrated PHREEQC-based saturation modelling with stable isotopes and multivariate statistical analyses in this area. This lack of integration has limited our understanding of water–rock interaction processes, mineral stability, and their implications for water quality. To address these gaps, this study seeks to tackle the following research questions:

1. What are the major hydrochemical characteristics of groundwater in Oye-Ekiti, and what do they reveal about dominant geochemical processes and water–rock interactions?
2. How do the δ^2^H–δ^18^O values of groundwater compare with regional and LMWLs, and what do these comparisons indicate about the sources of recharge and evaporation?
3. Do shallow hand-dug wells show significant differences from boreholes in terms of chemistry, isotopic enrichment, and mineral-saturation states? What processes contribute to these differences?
4. What is the overall suitability of groundwater for drinking based on the World Health Organization (WHO)/ Nigerian Industrial Standards (NIS) guide values, and what are the implications for local water resource management?

To answer these questions and align the study objectives with testable outcomes, three falsifiable hypotheses (H_1_–H_3_) were formulated based on previous isotopic and hydrochemical investigations in the Ekiti Basement Complex and adjacent Dahomey Basin^10,31^. H_1_: Groundwater δ^2^H–δ^18^O values plot close to the regional LMWL with d-excess between 8 and 12‰, implying limited pre-infiltration evaporation. H_2_: Silicate weathering is the dominant geochemical process controlling major-ion composition, reflected in rock-dominance on the Gibbs diagram and yielding undersaturation with respect to carbonate minerals. H_3_: Hand-dug wells show greater evaporative enrichment than boreholes due to shallower depth and longer exposure to surface conditions during the dry season. These hypotheses provide a framework for quantitatively linking isotopic, chemical, and geochemical modelling results, thereby strengthening process-based interpretations of groundwater evolution in Oye-Ekiti.

This study represents the first comprehensive, dry season, city-scale geochemical investigation of groundwater quality and hydrogeochemical processes in Oye-Ekiti with the following specific objectives: (1) determine the groundwater major ions chemistry, (2) identify the dominant hydrochemical facies, (3) explore the mechanisms of hydrochemical evolution and ion-exchange, (4) characterize the δ^18^O and δ^2^H isotopic composition of groundwater from boreholes and hand-dug wells within the Crystalline Basement Groundwater System (CBGS) in Oye-Ekiti, and (5) compare the δ^18^O–δ^2^H groundwater regression line with the regional LMWL to determine the source and nature of groundwater recharge. The hydrogeochemical analysis encompasses descriptive statistics, Spearman’s correlation analysis, and multivariate statistics, along with advanced environmental geochemistry tools such as PHREEQC geochemical modeling software.

Groundwater stable isotopes (δ^18^O and δ^2^H), together with deuterium excess (d-excess), will be evaluated against the Global Meteoric Water Line (GMWL; δ^2^H = 8·δ^18^O + 10) as well as local and regional LMWLs to infer the origin, recharge processes, and isotopic evolution of the groundwater. The isotopic composition of groundwater samples which plot close to the regional LMWL and have d-excess values around 10‰, indicates that recharge likely occurred from precipitation with minimal evaporation before infiltration, suggesting direct or rapid percolation through the unsaturated zone under humid or moderate climatic conditions.

The present study introduces the first city-scale dataset for Oye-Ekiti that integrates δ^18^O–δ^2^H data with PHREEQC-based saturation indices, multivariate statistical analysis, and major-ion chemistry within a unified framework. Previous isotope studies, including those of Talabi and Tijani^10^ in the Ekiti basement, Aladejana et al.^31^ in the Eastern Dahomey Basin, and Yusuf et al.^30^ in the Lagos coastal aquifer, provided valuable regional insights, but were conducted at broader spatial scales and under mixed or wet-season conditions. In contrast, this research focuses on an urban crystalline basement setting during the dry season, offering higher spatial resolution and the first integration of isotopic and thermodynamic geochemical modelling for Oye-Ekiti. This approach establishes a new geochemical and isotopic baseline, which can help inform groundwater management strategies and enhance our understanding of crystalline basement aquifers in southwestern Nigeria.

## Study area

### Location and description of the study area

**Fig. 1:**
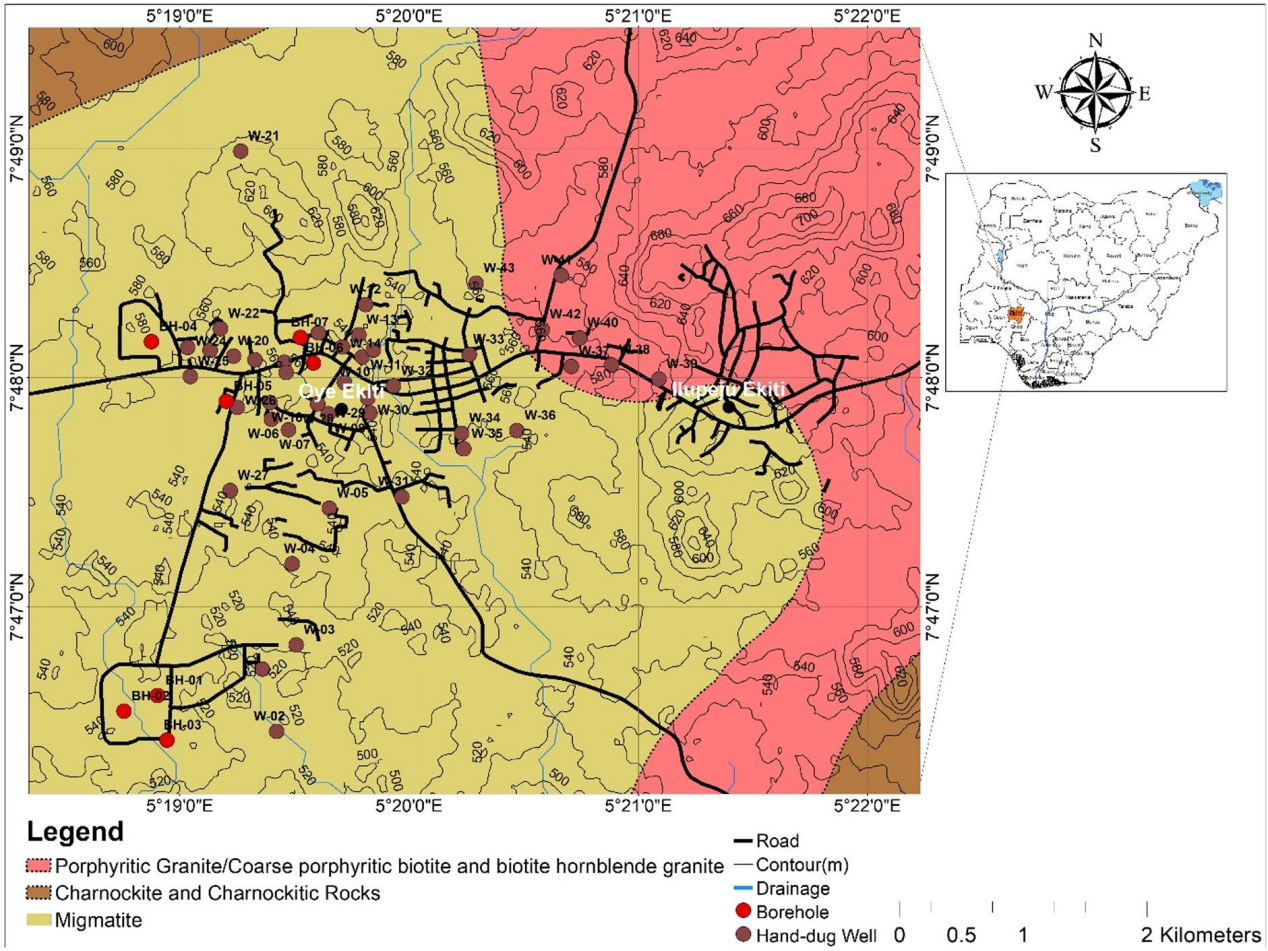
Location and geological map of the study area showing sampling points. (The map was generated using ArcGIS Pro Desktop version 3.0; URL: https://www.esri.com/en-us/arcgis/products/arcgis-pro/overview).

Oye-Ekiti is located between latitudes 7° 46′20′′N and 7° 48′40′′N, and longitudes 5° 18′40′′ E and 5° 21′ 20′′ E within the rainforest belt of southwestern Nigeria (Fig. 1). The spatial distribution of sampling points and the geological framework presented in Fig. 1 were mapped and processed using ArcGIS Pro Desktop (v. 3.0). The topography is predominantly low-lying to undulating, with elevations exceeding 500 m. The southern part is characterized by plains, while the northern part features gently sloping to steep-sided outcrops forming a west–east trending ridge. The area has a tropical climate with alternating wet and dry seasons^10^ and is drained by river networks exhibiting a dendritic pattern, including seasonal tributaries and incipient streams (Fig. 1). The rainy season typically occurs from around April to October, peaking in September with heavy rainfall, while the dry season extends from November to March and is characterized by the dry, dust-laden north-east trade wind which is popularly known as harmattan^32^. Oye-Ekiti has a dendritic drainage pattern and receives roughly 140 mm (5.55 inches) of precipitation annually with an average of 238.54 rainy days (65.35% of the year) annually^32^. The average relative humidity is 73.43%, and the mean annual temperature ranges from 21.78 to 32.69 °C (71.20–90.84 °F)^32^.

According to the National Population Commission (NPC) website, Oye-Ekiti had a population of approximately 137,796 in 2006, which increased to 206,300 by 2022 (NPC^95^). This population growth is mainly attributed to the establishment of the Federal University Oye-Ekiti in 2012. The resulting influx of university staff and students has significantly affected the town’s amenities, particularly water supply and sanitation services.

### Geological setting and hydrogeologic framework

The Oye-Ekiti area is located on Precambrian crystalline basement (metamorphic and igneous) rocks^9,10,33^. Granitic rocks are younger than metamorphic rocks in the study area. The main lithologies include coarse and medium-grained charnockites, granites, granite gneiss, banded gneiss, and migmatites (Fig. 1). These migmatites and gneisses occur as migmatite gneiss, granite gneiss and banded gneiss. The age of the migmatite gneiss complex has been found to be between 2.0 and 3.0 Ga^33–35^. These gneisses and migmatites can be found as banded gneiss, granite gneiss, and migmatite gneiss. It has been shown that the migmatite gneiss complex is 2.5–3.0 Ga old^33–35^. Charnockites and granites are Pan-African (600 ± 150 Ma) in age and are later intrusions within the migmatite–gneiss–complex^35–37^. Charnockites and migmatite contacts are located along the boundary between Oye-Ekiti and Ilupeju; however, observed grain size increased away from the contact zone. Weathering is more pronounced in the coarse-grained charnockites around Ilupeju, with large granitic grains, compared with the fine to medium-grained charnockites and granites. The charnockites are greenish to dark grey, and mostly occur at the margin of the older granitic bodies^35,38,39^.

The hydrogeological settings of Oye-Ekiti could be described in terms of the lithology, structures, and discontinuities that govern the occurrence, amount, flow and groundwater chemistry^19^. Oye-Ekiti relies on secondary porosity and its associated permeability for groundwater potentials. Basement complex rocks seldom possess primary porosity, making them poor to average aquifers. However, through the effects of a series of orogenic and other tectonic activities, secondary porosities and permeability are common as a result of the presence of fractures, faults, joints, shearing, fissures, and intense weathering^9,10^. Most of the groundwater in the area is found in regoliths and saprolites, which are rocks that have weathered partially or completely^9^. Depending on the lithology and mineralogy of the rocks, there are variations in the thickness of the overburden (regolith), and it is found that this fluctuation is what causes the variation in groundwater potentials at various locations, particularly within Nigeria’s basement complex^10^. Based on field observation, the peculiar hydrogeologic settings of Oye-Ekiti and its environs are characterized by localized aquifers mostly found within the regolith and saprolite units. The aquifer system of the area is mostly semi-confined to unconfined aquifers, like most places in the southwestern region of Nigeria^9,10^.

## Materials and methods

### Field sampling

A total of 50 water samples were collected from sources used for domestic purposes in the study area: seven (7) boreholes and 43 hand-dug wells, for physicochemical and environmental isotope analyses. Sampling locations were selected based on the availability and accessibility of domestic water sources between January and February 2022, as shown in Fig. 1, following American Public Health Association (APHA^98^) and International Atomic Energy Agency (IAEA^99^) protocols. Hand-dug wells are typically shallow, while records of well completion for the boreholes were unavailable. Polyethylene sampling bottles (750 mL) were pre-treated by repeatedly washing with deionized water and drying at room temperature prior to sample storage. Before sample collection, the boreholes were allowed to pump for about 10 min to remove residual water, and samples were collected at the wellhead. Samples from the hand-dug wells were collected by lowering sampling buckets into each well, with depths measured using a Temperature-Level-Conductivity (TLC) meter. Boreholes were allowed to pump for about 30 min before sample collection, while hand-dug wells could not be purged conventionally due to their large open diameter and the risk of wasting the scarce resource during the dry season. We discarded the first draw-off, and samples from water wells were collected after allowing the water column to settle, following APHA procedures for traditional wells. To ensure that reported concentrations represent the dissolved phase, all groundwater samples intended for major-ion and nutrient analyses were filtered in the field through 0.45 µm membrane syringe filters immediately after collection. Samples for major cations were collected in pre-cleaned bottles, filtered, and acidified in the field to pH < 2 with ultrapure HNO_3_ within two hours of sampling to prevent precipitation, adsorption, or biological alteration. Samples for anions and nutrients (including PO₄^3^⁻ and NO₃⁻) were filtered but not acidified, and stored below 4 °C until analysis. At all sampling stations, samples were collected in duplicates, with each bottle completely filled and tightly covered at the collection points. Sample bottles were labeled appropriately and securely stored in ice-tight coolers for onward transmission to the laboratory for analysis. Immediately after sampling, *in-situ* measurement of pH, Eh, Electrical Conductivity (EC), Resistivity, Total Dissolved Solids (TDS), Salinity, Temperature, and Pressure were determined in the field using the Hanna Instruments digital pH-multiparameter meter (HI98194). Measurements were taken after the readings stabilized, and reference coordinates were recorded by means of the Garmin 76 handheld Global Positioning System (GPS). The dimensionless coefficient of variation (CV) has been used to characterize the spatial scale variation in hydrogeological variables and to measure sample variability without accounting for data scale. It could provide insights into the factors that affect the spatial distribution of the major elements. Minor (weak) variation is indicated by a CV between 0 and 10%, medium variation by a CV between 10 and 100%, and large (strong) variation by a CV greater than 100%.

### Sampling design and potential bias

Groundwater samples were collected using a convenience sampling approach based primarily on site accessibility, availability of operational wells, and permission from well owners. This approach was adopted because a comprehensive registry of groundwater abstraction points in the study area was unavailable and many wells are privately owned. Consequently, sampling locations were selected from accessible hand-dug wells and boreholes distributed across Oye-Ekiti. Specific depth information and construction records were not available during sampling for boreholes; however, typical depth ranges were inferred from secondary datasets obtained from the Ekiti State Rural Water Supply and Sanitation Agency and published studies^10^, (Bayowa et al.^100^). Accordingly, hand-dug wells generally represent the shallow unconfined weathered regolith aquifer (3–15 m), while boreholes tap the deeper fractured crystalline basement aquifer (30–80 m). These depth ranges represent nominal estimates derived from regional operational records and previously published hydrogeological studies; however, individual well depths at the sampled locations could not be confirmed in field records due to limited access to construction data for privately installed wells. Because sampling locations were partly selected based on accessibility, the dataset may preferentially represent groundwater conditions within urban and peri-urban zones, potentially underrepresenting hydrochemical characteristics of rural aquifer conditions in the surrounding areas. This potential sampling-frame bias should therefore be considered when interpreting spatial patterns and extrapolating the results beyond the sampled locations.

### Chemical and stable isotope analyses

Groundwater samples were analyzed for the following dissolved chemical constituents: calcium (Ca^2+^), magnesium (Mg^2+^), sodium (Na^+^), potassium (K^+^), sulfate (SO_4_^2−^), nitrate (NO_3_^–^), chloride (Cl^–^), and bicarbonate (HCO_3_^–^) ions using standard analytical methods recommended by the American Public Health Association (APHA 2017) as described by Bolaji et al.^40^. Bicarbonate (HCO_3_^-^ was determined using the acid–base titration method; NO_3_^-^ was measured by the UV spectrophotometric method at a wavelength of 220 nm with correction at 275 nm (APHA 4500-NO_3_^–^); Cl^–^ analysis was carried out by AgNO_3_ titration; sulfate was analyzed using the turbidimetric BaCl_2_ method, measured spectrophotometrically (APHA 4500-SO_4_^2–^ E), while phosphate (PO_4_^3-^) was determined using the ascorbic acid method (APHA 4500-P) and measured colorimetrically; TH was determined by EDTA titration, while TDS was measured using gravimetric method. Sodium, K^+^, Ca^2+^, and Mg^2+^ were determined using the Flame Atomic Absorption Spectroscopy (AAS), equipped with element-specific hollow cathode lamps, following (APHA 3111B/D) procedures with analytical wavelengths 589.0 nm, 766.5 nm, 422.7 nm, and 285.2 nm, respectively. We ensured quality assurance and quality control (QA/QC) as average values of each data obtained from chemical analysis were reported from duplicate measurements. The ionic balance method (Eq. 1) was used to verify raw data. Although certain samples exhibited charge balance errors (CBEs) exceeding the commonly accepted threshold of ± 5%, these elevated CBE values primarily occurred in low TDS waters, where analytical uncertainties are inherently higher due to low ionic strength. Charge balance error is defined as the relative difference between the sum of cations and anions. It is a standard quality control metric in hydrogeochemistry. However, low ionic strength waters naturally tend to have larger proportional errors; this does not necessarily indicate analytical invalidity^41,42^. Accordingly, leading hydrogeochemical references recommend retaining samples with moderate CBEs when they are consistent with regional hydrogeologic trends and other chemical indicators^43–45^. In this study, although some samples have CBE values below -5%, their chemical signatures align with expected hydrogeologic patterns and do not contradict other analyses, supporting their inclusion. Excluding these samples would yield a less representative dataset, with little improvement in overall reliability, since systematic laboratory errors were minimized through standard quality control procedures. Thus, maintaining these samples ensures comprehensive representation of the hydrochemical variability inherent in the study region, consistent with best practices in groundwater quality assessment.

1 $$CBE=\left[\frac{\sum Cations- \sum Anions}{\sum Cations+ \sum Anions}\right]\times 100$$

Samples for δ^2^H and δ^18^O analysis were collected in airtight 20 mL amber-glass vials with Teflon-lined caps. Each vial was rinsed three times with sample water and filled without headspace, following IAEA recommendations. Groundwater samples were analyzed for ^2^H/^1^H and ^18^O/^16^O ratios using a Picarro L2130i liquid water isotope analyzer via cavity ringdown spectroscopy (CRDS) at the University of Dayton water isotope lab. Results are reported in the δ-notation in permil (‰) relative to the international standard Vienna Standard Mean Ocean Water (VSMOW) convention:

2 $${\delta } \left(\permil \right)= \frac{{R}_{sample}-{R}_{standard}}{{R}_{standard}} \times 1000$$

where δ represents δ^18^O and δ^2^H, and R is the isotopic ratio ^2^H/^1^H and ^18^O/^16^O of a sample (R_sample_) and a standard (R_standard_). These data are discussed in terms of d-excess = δ^2^H—(8 × δ^18^O) of the Global Meteoric Water Line (GMWL)^46^. Repeated measurements of three commercially available internal standards calibrated to the VSMOW–Standard Light Antarctic Precipitation (SLAP) scale, PZ (δ^18^O = 0.3‰, δ^2^H = 1.8‰), PM (δ^18^O = − 20.6‰, δ^2^H = − 159.0‰), and PD (δ^18^O = − 29.6‰, δ^2^H = − 235.0‰), were used as internal and external references to calibrate the raw isotope measurements. To ensure consistency, each analytical run consisted of five samples bracketed by standards and typically yielded standard deviations of 0.1‰ for δ^18^O and 0.4‰ for δ^2^H. The instrument was periodically checked for drift. Each sample was analyzed with 6–9 injections, and the first three injections were discarded to minimize memory effects. Finally, analytical results were further examined for potential organic interference using the Picarro ChemCorrect™ software (version 1.2.1) (https://www.picarro.com/support/software_downloads).

### Hydrochemical data analysis

Hydrochemical facies were identified using Piper and Durov diagrams to classify groundwater types and infer geochemical evolution. The contributions of anthropogenic activities and water–rock interaction to groundwater can be understood by hydrochemical processes^47^. Precipitation, rock weathering, and evaporation are the main processes that control the dissolved chemical ion constituents^48^. Gibbs provided two diagrams showing the weight ratios of Cl^−^/(Cl^−^ + HCO_3_^−^) (x-axis) and Na^+^/(Na^+^ + Ca^2+^) (x-axis) against TDS (y-axis) in order to determine the functional contributors to dissolved chemicals. Bivariate ionic relationships and molar ratio plots (e.g*.*, Na^+^/Cl⁻, Ca^2+^ + Mg^2+^ *vs.* HCO_3_⁻ + SO_4_^2^⁻) were used to assess the roles of silicate weathering, carbonate dissolution, and cation exchange processes. Chloro-alkaline indices were calculated to further evaluate ion exchange reactions between groundwater and aquifer materials. Cation exchange is an essential geochemical process that significantly affects the evolution of the hydrochemical characteristics of natural waters^40^. Two chloro-alkaline indices, CAI_1_ and CAI_2_, were calculated in order to examine the influence of cation exchange on the groundwater evolution mechanism (Eqs. 3 and 4). All values are reported in meq/L.

3 $${\mathrm{C}\mathrm{A}\mathrm{I}}_{1} = \frac{{\mathrm{C}\mathrm{l}}^{- }- \left({\mathrm{N}\mathrm{a}}^{+ }+ {\mathrm{K}}^{+}\right)}{{\mathrm{C}\mathrm{l}}^{-}}$$

4 $${\mathrm{C}\mathrm{A}\mathrm{I}}_{2}= \frac{{\mathrm{C}\mathrm{l}}^{- }- \left({\mathrm{N}\mathrm{a}}^{+ }+ {\mathrm{K}}^{+}\right)}{{\mathrm{S}\mathrm{O}}_{4}^{2-}+ {\mathrm{H}\mathrm{C}\mathrm{O}}_{3 }^{-}+ {\mathrm{C}\mathrm{O}}_{3 }^{-}+ {\mathrm{N}\mathrm{O}}_{3}^{-}}$$

When K^+^ and Na^+^ ions in water exchange with Ca^2+^ and Mg^2+^ ions in rock, reverse-ion exchange is indicated by positive chloro-alkaline indices (CAIs). The reverse-ion exchange process, on the other hand, occurs when the CAI value is negative and indicates a direct ion-exchange reaction between Ca^2+^ and Mg^2+^ in water and Na^+^ and K^+^ in rocks^49,50^.

### Geochemical modeling

Using the thermodynamic database MINTEQv.4 and the geochemical software—PH REdox EQuilibrium in C language (PHREEQC) version 3.8.6, an Open Source Software (OSS) developed by the United States Geological Survey (USGS)^51^ https://www.usgs.gov/software/phreeqc-version-3), the saturation indices (SIs) of relevant mineral phases were computed to assess groundwater chemical reactions^52^. The MINTEQv.4database was selected because it provides a detailed representation of aqueous speciation, mineral equilibria, and surface complexation processes relevant to groundwater, and is widely used in hydrogeochemical studies (e.g^40,53,54^., Nwankwoala et al.^101^), ensuring robust and comparable results. Hydrogeochemical processes are governed by the water–rock interaction process, with geochemical modeling conducted to derive SIs for the identified mineral phases in accordance with Eq. (5):

5 $$\mathrm{S}\mathrm{I}=\mathrm{l}\mathrm{o}\mathrm{g} \left(\frac{\mathrm{I}\mathrm{A}\mathrm{P}}{{\mathrm{K}}_{\mathrm{s}\mathrm{p}}}\right)$$

where K_sp_ is the equilibrium constant, and IAP is the Ion Activity Product. When the SI value is equal to zero, the solution is in equilibrium with the mineral phase; when the SI value is greater than zero, mineral precipitation occurs; and when the SI value is less than zero, dissolution is required to achieve equilibrium, indicating that the solution is undersaturated^54^.

### Statistical analyses

#### Spearman’s correlation coefficients

The relationship between two parameters was examined using correlation analysis, which took into consideration all data samples, including outliers, due to sample size limitations. To mitigate the potential impact of outliers, the non-parametric Spearman rank correlation coefficient (r) was employed, providing a reliable statistic that is less susceptible to extreme results^55^. The following formula was used to determine the Spearman’s rank correlation coefficient (6)^56^:

6 $$r_{s} = 1 - \frac{{6\sum\limits_{i = 1}^{n} {d_{i}^{2} } }}{{n(n^{2} - 1)}}$$

where n = number of cases, x_i_ and y_i_ = data pairs, and d_i_ = difference in paired ranks.

When ranks are tied, the formula to use is (7):

7 $$\rho = \frac{{\sum\nolimits_{i} {(x_{i} - \overline{x} )} (y_{i} - \overline{y} )}}{{\sqrt {\sum\nolimits_{i} {(x_{i} - \overline{x} )^{2} \sum\nolimits_{i} {(y{}_{i} - \overline{y} )^{2} } } } }}$$

Using correlation coefficient values ranging from − 1 to 1, five different categories were established based on the magnitude of their absolute values. These categories showed the following levels of association strength: very strong (0.80–1.00), strong (0.60–0.79), moderate (0.40–0.59), weak (0.20–0.39), and very weak (0.00–0.19)^57^. This classification reflects a gradient from the strongest to the weakest associations. Based on p-value criteria, the correlation coefficient’s statistical significance is evaluated as follows: not significant at *p* > 0.10, moderately significant at *p* < 0.05, and highly significant at *p* < 0.01. All statistical computations, including Spearman’s correlation, Factor Analysis (FA), and Hierarchical Cluster Analysis (HCA), were performed using IBM SPSS Statistics version 26 (https://www.ibm.com/products/spss-statistics). Data organization, table preparation, and initial plotting were conducted using Microsoft Excel 2021 (https://www.microsoft.com/en-us/microsoft-365/excel).

#### R- and Q-modes hierarchical cluster analysis

Hierarchical Cluster Analysis (HCA) is a multivariate statistical method used to group individuals with comparable characteristics. In hydrogeochemical research, samples (Q-mode) or parameters (R-mode) are organized into clusters based on their degree of (dis)similarity, which are graphically depicted in a dendrogram. In the present study, the HCA was performed using an Aitchison-appropriate distance, calculated as Euclidean distances on centered log-ratio (CLR)–transformed data, with Ward’s linkage method (1963). To assess the robustness of this approach, a sensitivity analysis was conducted by varying the linkage method and by repeating the analyses with and without samples identified as compositional outliers in CLR space.

#### Factor analysis

Factor analysis (FA) is a valuable method for examining the interrelationships among numerous parameters and understanding the underlying influences on them. This method is used to evaluate complex datasets, reduce dimensionality by limiting the number of parameters, and construct new subsets, termed factors, that group similar parameters. To determine the factors, the dataset was subjected to Principal Component Analysis (PCA), which utilized Varimax rotation with Kaiser normalization^58^. We applied the CLR transformation to our compositional dataset. Log-ratio transformations, such as CLR, address the constant-sum constraint characteristic of compositional data by transforming raw measurements (*e.g.*, geochemical data) into log-ratios. This approach eliminates spurious correlations and enables valid statistical analyses in Euclidean space^59^. Varimax rotation maximizes the variance between factors by using an orthogonal transformation matrix for enhanced interpretability. Each factor’s eigenvalue reflects its significance,according to the Kaiser criterion, only factors with eigenvalues ≥ 1.0 are retained^60^, factors with eigenvalues less than this were not included in the analysis. To better understand the relationships between parameters, factor loadings were categorized into three different groups.

Based on the absolute values, three categories have been identified: weak (0.30–0.50), moderate (0.50–0.75), and strong (0.75–1.00)^61^. The Kaiser–Meyer–Olkin (KMO) measure of sampling adequacy^62^ and Bartlett’s test of sphericity^63^ were used to assess the suitability of the factor analysis (FA) approach and the sufficiency of the dataset.

## Results and discussion

All groundwater samples analyzed in this study were collected during the dry season. Consequently, the reported solute concentrations, stable-isotope compositions (δ^18^O and δ^2^H), and interpretations related to evaporative enrichment and groundwater evolution reflect dry-season hydrochemical conditions and may not represent the full annual variability of groundwater chemistry in the study area.

### Hydrochemical characteristics

**Fig. 2:**
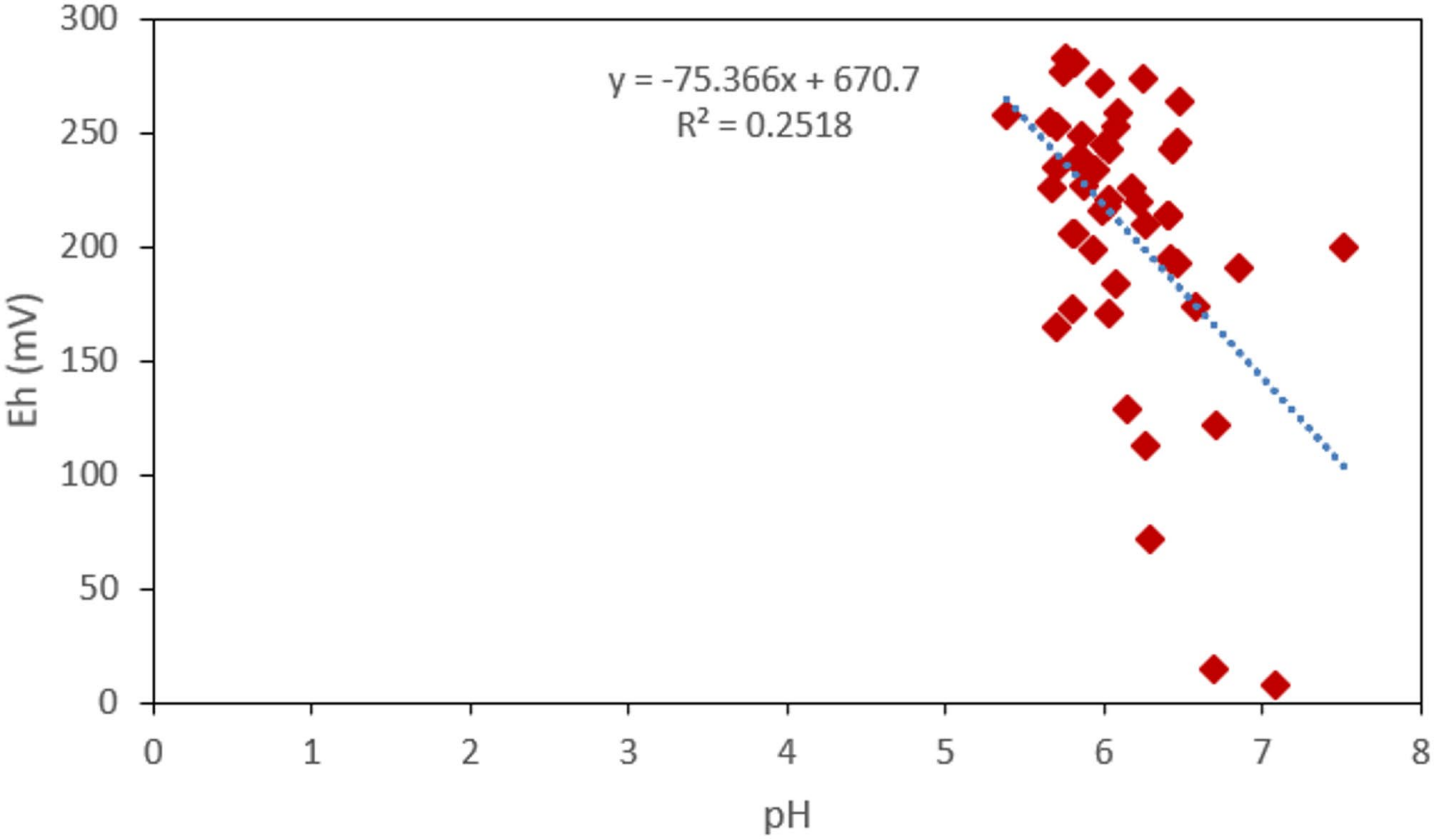
Bivariate relationship of pH vs. Eh of the studied groundwater samples from the Oye-Ekiti area.

**Fig. 3:**
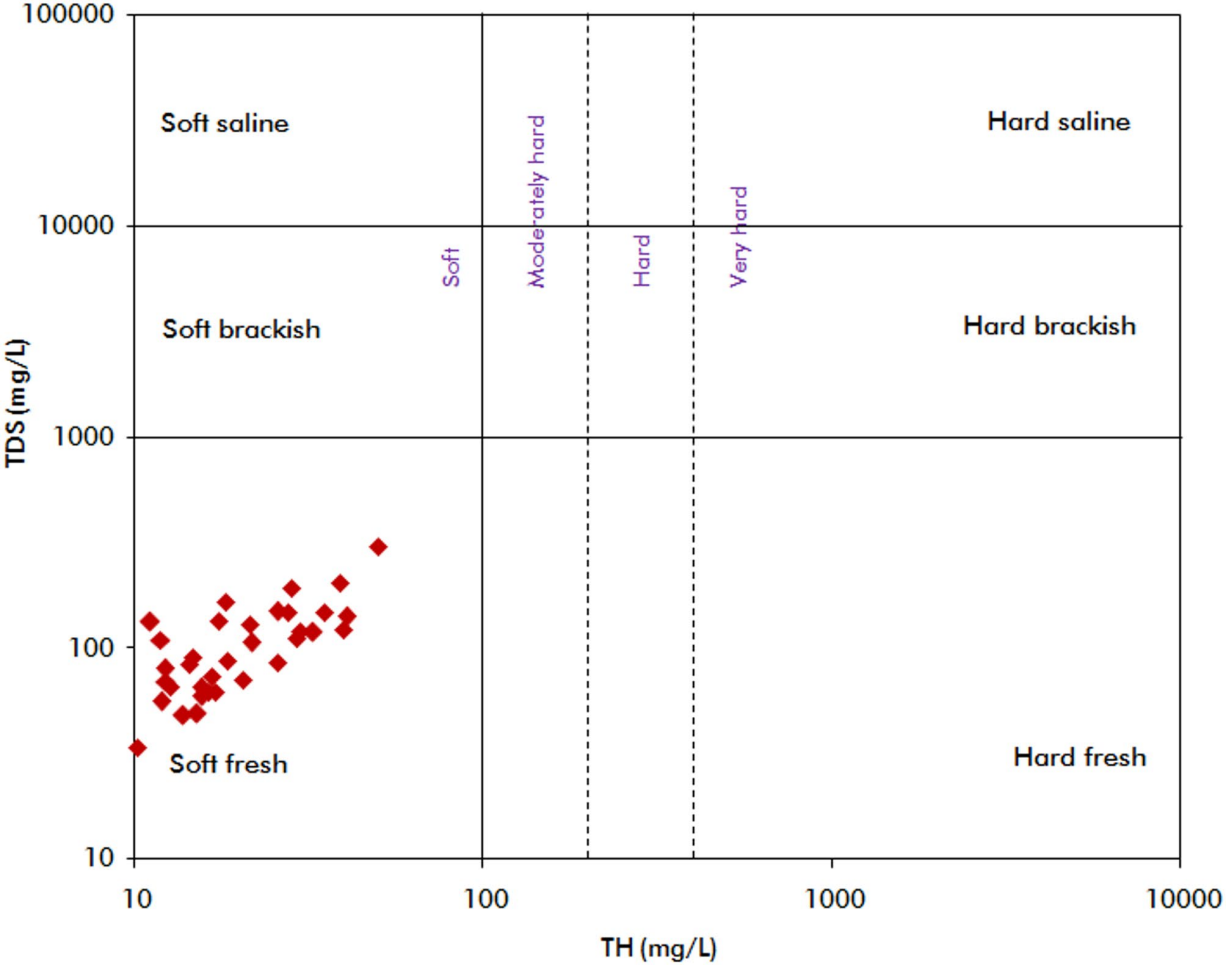
Total Dissolved Solids (TDS) vs*.* Total Hardness (TH) scatterplot of the studied groundwater samples from the Oye-Ekiti area, showing the different fields of classification.

Table 1 presents the descriptive statistics for the groundwater samples investigated. The samples’ temperatures fall between 24.92 and 29.92 °C. The measured pH values range from 5.39 to 7.52, indicating acidic to slightly alkaline water, with the majority of results (88%) falling below the recommended pH range (i.e., from 6.5 to 8.5) for drinking^64,65^. These acidic waters would naturally enhance the chemical weathering process in the area by dissociating Na-feldspars and other susceptible minerals into clay minerals such as kaolinite^66^, Eq. 8). Although the underlying geology of Oye-Ekiti is dominated by migmatite-gneiss and granitic rocks, the thick tropical regolith developed over these lithologies commonly contains secondary clay minerals, such as kaolinite, formed by feldspar weathering. Redox potential (Eh) ranges from 8.20 to 282.5 mV, EC ranges between 49.0 and 607.0 µS/cm, while TH ranges from 1.4 to 50.1 mg/L. A weak positive relationship exists between the pH and Eh in the studied groundwater samples (Fig. 2). The water resistivity measured *in-situ* ranges from 0.0016 to 0.0204 MΩ^−1^ cm, whereas TDS values range between 24 and 304 mg/L. Water salinity is generally very low (0.09‰), while pressure ranges from 13.91 to 14.16 psi, respectively. Based on the classification scheme for groundwater hardness, the studied samples are soft, freshwater (Fig. 3).

**Table 1:** Descriptive statistics (Min. = Minimum value, Max. = Maximum value, Mean = average value, Median = Median value, Std Dev. = Standard Deviation) of the physicochemical parameters and environmental isotopes in groundwater samples (N = 50) from the Oye-Ekiti area, compared with World Health Organization^65^ standard and the Nigerian Standard for Drinking Water Quality^64^.

Parameters	Units	Groundwater (N = 50)							
		Min	Max	Mean	Median	Std Dev	CV (%)	WHO^65^	NIS^64^
pH	–	5.39	7.52	6.12	6.04	0.40	6.53	6.5–8.5	6.5–8.5
Temp	°C	24.92	29.92	26.72	26.77	0.79	2.96	30	Ambient
Eh	mV	8.20	282.50	209.37	223.30	60.05	28.68	–	–
EC	µS/cm	49.00	607.00	188.90	162.00	112.87	59.75	–	1000
TH	mg/L	1.40	50.10	16.31	14.50	11.67	71.56	–	150
Ca	mg/L	4.01	28.89	16.16	16.03	6.84	42.32	–	–
Mg	mg/L	0.15	10.82	3.63	3.18	2.58	70.97	–	–
Na	mg/L	1.00	76.00	22.02	19.50	14.16	64.31	200	200
K	mg/L	1.00	21.00	6.46	6.00	3.88	60.09	–	–
HCO_3_	mg/L	20.40	165.60	58.78	52.25	28.49	48.48	–	–
Cl	mg/L	5.32	92.17	22.98	19.50	15.12	65.79	250	250
SO_4_	mg/L	5.22	68.20	22.60	14.12	19.08	84.42	250	100
NO_3_	mg/L	0.01	0.91	0.18	0.09	0.22	117.93	50	50
PO_4_	mg/L	3.04	32.52	10.58	10.89	3.96	37.47	–	–
TDS	mg/L	24.00	304.00	94.46	81.00	56.57	59.88	-	500
ẟ^18^O	V_SMOW_ (‰)	− 3.53	− 2.68	− 3.11	− 3.11	0.21	–	–	–
ẟ^2^H	V_SMOW_ (‰)	− 17.65	− 11.96	− 14.50	− 14.29	1.55	–	–	–
d-excess	V_SMOW_ (‰)	5.60	12.22	10.41	10.45	0.79	–	–	–

CV = Coefficient of variation.

8 $$2{NaAlSi}_{3}{O}_{8} + {2CO}_{2} + {11H}_{2}O \to {Al}_{2}{Si}_{2}{O}_{5}{\left(OH\right)}_{4} + {4H}_{4}{SiO}_{4} + {2HCO}_{3}^{-}$$

Table 1 shows the results of the descriptive chemical parameters, including environmental isotope data. Except for pH, all the other chemical parameters are within the Nigerian Standard for Drinking Water Quality^64^ and the World Health Organization^65^ regulatory limits. Hydrogeological conditions, topography, hydrometeorology, and anthropogenic activities are examples of external environmental factors that are likely to have impacted hydrochemical elements with CVs greater than 100%^67,68^. This indicates significant sensitivity to environmental shifts and reflects large spatial changes in groundwater composition^69^. Analysis of variability indicated that almost all parameters had very small, consistent CVs, suggesting that the natural environment has a greater influence on these ions than anthropogenic activities. With a CV higher than 100%, NO_3_ showed elevated overall spatial variability, indicating that both the natural environment and human activity have a significant impact on this ion. The predominantly agrarian land-use pattern near Oye-Ekiti includes the use of N-bearing fertilizers, agrochemicals, and organic manure, especially during the rainy season proceeding the sample period. Furthermore, there are numerous sources of NO_3_, both point and diffuse, due to the extensive use of on-site sanitation systems (pit latrines and septic soakaways) and insufficient setback distances from manually dug wells. Deeper boreholes typically show lower and more consistent NO_3_ concentrations due to longer residence times, dilution, and reduced connectivity with surface sources; shallow wells, which frequently lack adequate sanitary protection and tap the upper regolith aquifer, are particularly susceptible to such contamination. Given the agrarian nature of the study area, it may be deduced that the high CV of NO_3_ suggests that pesticides, fertilizers, or animal manure may have had a substantial impact on some of the sampling locations, potentially accounting for the large spatial variability^70^.

### Hydrogeochemical processes

#### Major ion distribution and hydrochemical facies

Based on the hydrochemical data from the study area, the concentration levels of the dissolved cations (Ca^2+^, Mg^2+^, Na^+^, and K^+^) and anions (HCO_3_^−^, Cl^−^, SO_4_^2−^, NO_3_^−^, and PO_4_^3−^) in the groundwater samples are generally within acceptable limits for human consumption and domestic purposes^65^ except for PO_4_^3−^, which ranges from 3.04 to 32.52 mg/L (median = 10.89 mg/L). Sodium is the dominant cation with concentrations ranging from 1.00 to 76.00 mg/L (median = 19.50 mg/L), followed by Ca^2+^ which ranges from 4.01 to 28.89 mg/L (median = 16.03 mg/L), while HCO_3_^−^ is the dominant alkalinity species in the groundwater, followed by Cl^−^ with values ranging from 5.32 to 92.17 mg/L (median = 19.50 mg/L). Given the measured pH range of 5.39–7.52 (with ~ 88% of samples < 6.5), negligible carbonate ions, and alkalinity resides almost entirely as HCO₃⁻, consistent with APHA and USGS carbonate-equilibrium principles. The relative abundance of these ions is as follows: Na^+^ > Ca^2+^ > K^+^ > Mg^2+^, and HCO₃⁻ > Cl⁻ > SO₄^2^⁻ > NO₃⁻ > PO₄^3^⁻ on average.

**Fig. 4:**
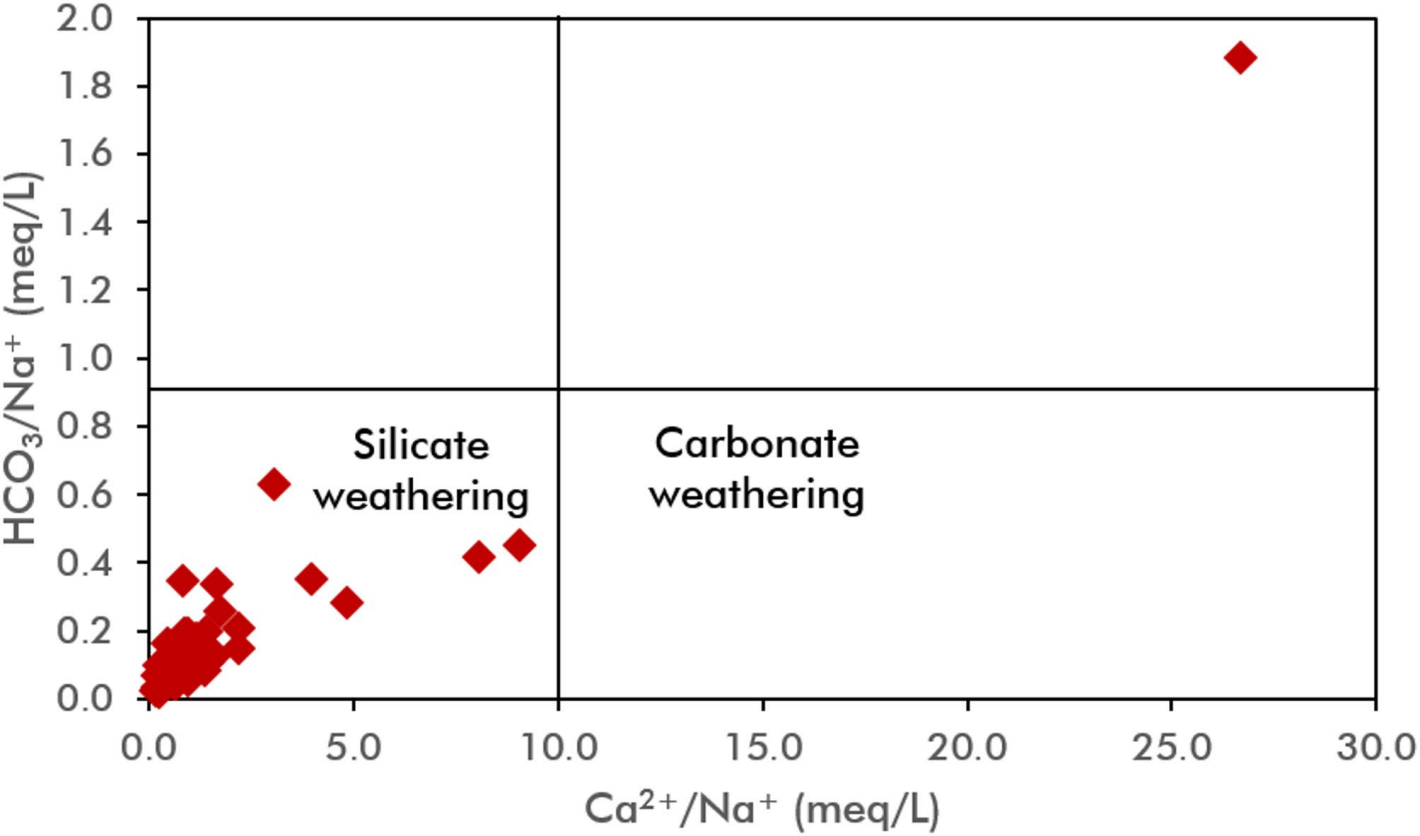
Bivariate relationship between HCO_3_/Na and Ca/Na of the studied groundwater samples from the Oye-Ekiti area.

In the studied samples, most of the ions (Na^+^, K^+^, Ca^2+^, Mg^2+^, HCO_3_^−^, and Cl^−^) showed statistically significant moderate to strong (i.e., TH, Mg^2+^, Na^+^, HCO_3_^−^; r > 0.5) and weak to moderate (i.e., Ca^2+^, Cl^−^; r < 0.5) positive correlations with TDS (Table 3), suggesting that their concentration has been impacted by chemistry of the flow path and water–rock interaction within the aquifer system. In a Precambrian crystalline basement terrain dominated by migmatite–gneiss and granitic rocks, where water–rock interaction is predominantly controlled by silicate weathering rather than carbonate dissolution (Fig. 4), the observed hydrochemical features are compatible with groundwater circulation. The hydrolysis of feldspars and ferromagnesian minerals, which releases Ca^2+^, Mg^2+^, Na^+^, and HCO_3_⁻ into solution, is the primary way that groundwater chemistry evolves in these basement aquifers. Carbonate minerals are usually absent or only present as minor secondary phases. Because bicarbonate is produced by CO_2_-driven silicate weathering rather than carbonate mineral dissolution, this geochemical framework explains the preponderance of Ca–Mg–HCO_3_ facies and the typically low alkalinity seen in the study area^10,44^. The observed pH values (5.39–7.52) are suggestive of the low buffering capacity of the silicate-dominated host rocks and their equilibration with soil CO_2_. Such pH levels tend to occur in basement aquifers where residence times are short, recharge is relatively recent, and the weathered regolith provides less alkalinity than sedimentary carbonate systems. Therefore, rather than being hypothetical or unusual, the geochemical signatures observed in the groundwater are primarily controlled by lithology and consistent with accepted concepts of groundwater evolution in hard-rock aquifers.

**Fig. 5:**
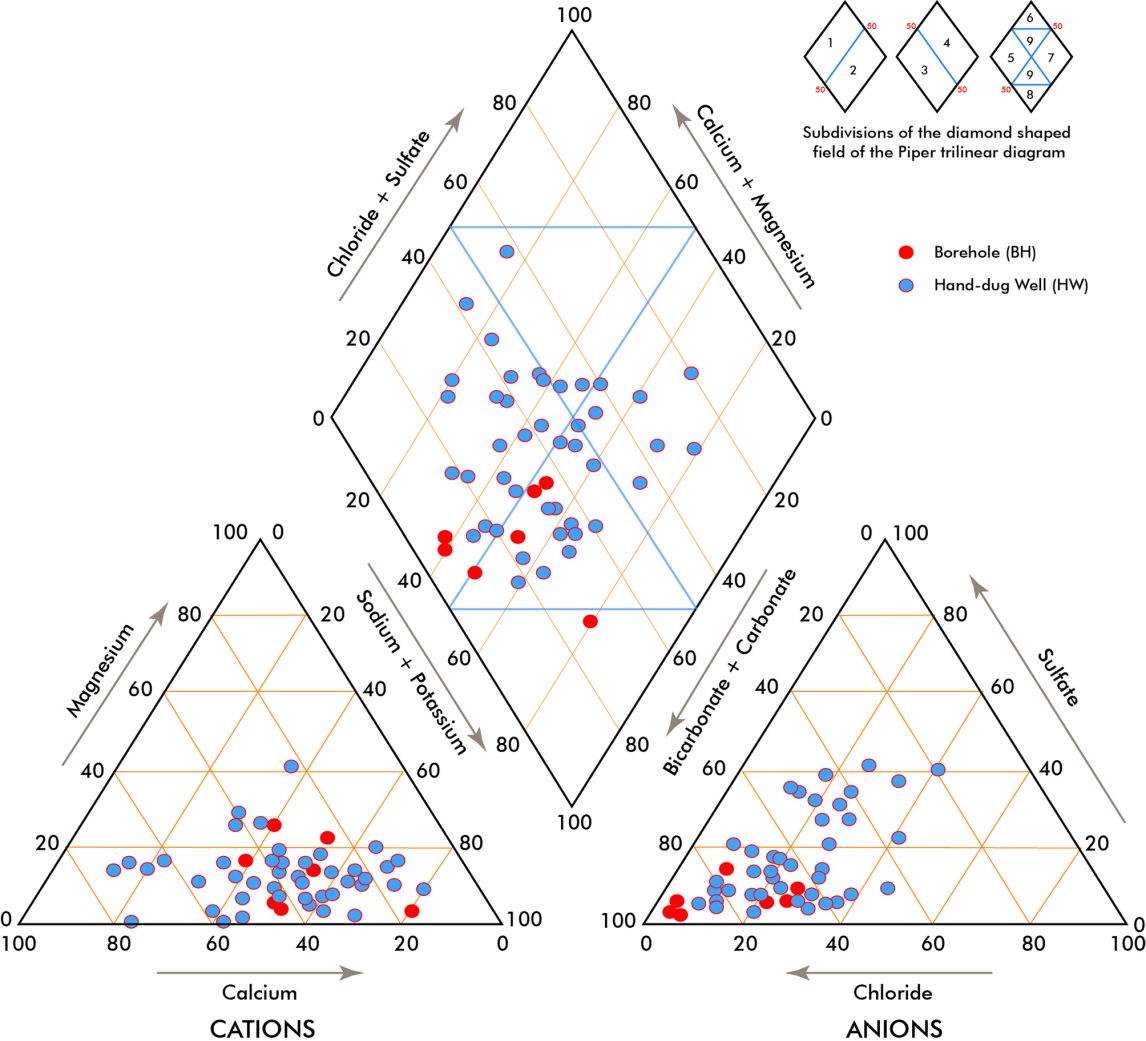
Piper diagram^71^ of major ion chemistry for groundwater samples from the Oye-Ekiti area.

Results of major ion chemical analyses were plotted on a Piper diagram for the collected samples^71^ (Fig. 5). According to the Piper diagram, the predominant groundwater facies in the study area (about 44% of samples) is Ca–Mg–HCO_3_, which is compatible with recharge-controlled groundwater in a crystalline basement aquifer. This facies reflects short residence times and limited geochemical evolution, with groundwater chemistry primarily governed primarily by silicate weathering reactions in migmatite–gneiss and granitic host rocks rather than by carbonate dissolution, absent in the local geology^43,44^. The remaining samples plot as mixed (non-dominant) water types, showing transitional compositions driven by varying flow patterns and degrees of water–rock interaction in the fractured bedrock and weathered regolith. A few samples fall within the Na–Cl and Na–HCO_3_ facies, representing localized, more evolved hydrochemical conditions, potentially related to longer residence times and cation exchange processes, but these facies are not regionally dominant. Generally, the Piper diagram demonstrates a groundwater system primarily influenced by active recharge, with minor spatial heterogeneity that reflecting variability at the aquifer scale rather than extensive geochemical evolution.

#### Rock weathering

**Fig. 6:**
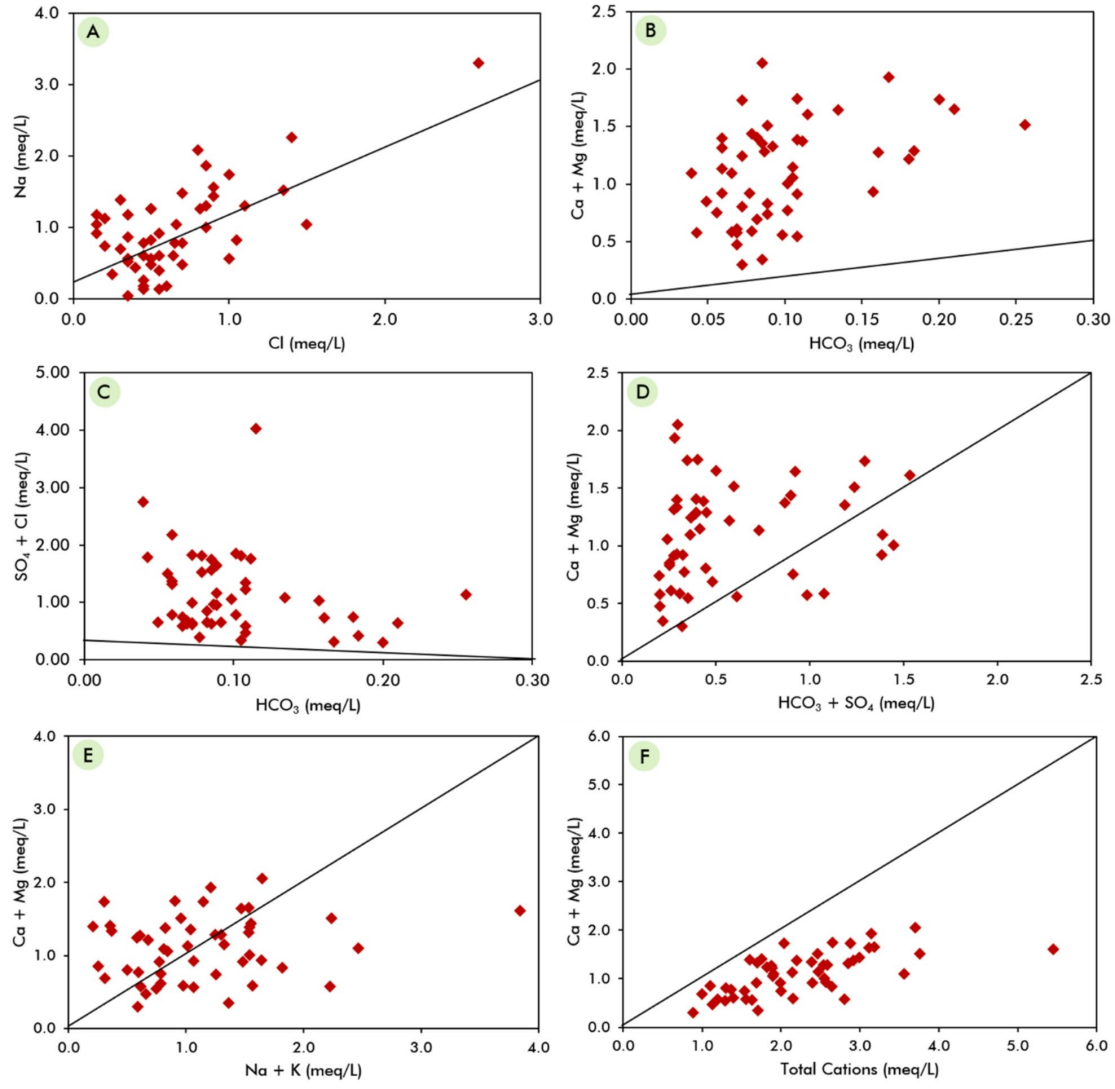
Bivariate ionic relationships between water quality variables: (**A**) Na vs. Cl; (**B**) Ca + Mg vs. HCO_3_; (**C**) SO_4_ + Cl vs. HCO_3_; (**D**) Ca + Mg vs. HCO_3_ + SO_4_; (**E**) Ca + Mg vs. Na + K; and (**F**) Ca + Mg vs. Total Cations.

If the system’s Na^+^ and Cl^−^ ions came from the dissolution of halite, the ratio of these ions would be equal to one. Whereas excess Na^+^ over Cl^−^ indicates the prevalence of cation exchange or rock weathering, excess Cl^−^ over Na^+^ indicates the predominance of reverse-ion exchange^72,73^. The Na–Cl relationship shows a generally positive correlation, with most samples plotting above the 1:1 equiline (Fig. 6A). This indicates an excess of Na relative to Cl, suggesting that halite dissolution is not the sole source of sodium. Instead, Na enrichment is likely influenced by silicate weathering and/or cation exchange processes, in which Ca^2+^ and Mg^2+^ in solution are exchanged for Na⁺ adsorbed on clay mineral surfaces. The majority (74%) of the samples investigated are above the theoretical Na^+^ and Cl^−^ion lines, suggesting that either rock weathering or the cation exchange process is the primary source. A handful (26%) plotted below the Na^+^ and Cl^−^ ion equiline. In view of the presence of migmatite-gneiss and granitic rocks in the study area, and its tropical climatic conditions, the preponderance of the Na^+^ ion over the Cl^−^ ion can be regarded as having a geogenic origin. The limited number of samples close to the equiline may reflect minor contributions from anthropogenic inputs. The presence of Na-enriched groundwater cannot be explained by evaporite dissolution because halite is missing from the Precambrian crystalline basement underlying the study area. Rather, cation-exchange reactions between groundwater and clay-rich weathered regolith, where Ca^2+^ and Mg^2+^ in solution are exchanged for Na^+^ adsorbed on mineral surfaces, and prolonged interaction with Na-bearing silicate minerals are more likely to account for elevated Na^+^ relative to Ca^2+^ and Mg^2+^ in a small number of samples (Eqs. 9 and 10).

9 $$\left({Na}^{+}, {K}^{+}, {Ca}^{2+}, {Mg}^{2+}\right) Silicates+ {H}_{2}{CO}_{3} (Rock weathering) \to {Na}^{+}+ {K}^{+}+ {Ca}^{2+}+ {Mg}^{2+}+ {HCO}_{3}^{-}+ {H}_{4}{SiO}_{4}+Clay products$$

10 $$NaCl \left(Halite dissolution\right)\to {Na}^{+}+ {Cl}^{-}$$

The diagram ${Ca}^{2+}+ {Mg}^{2+}$ *vs.* ${HCO}_{3}^{-}$ is often used to confirm the dominance of cation exchange or rock weathering as controls on groundwater quality^74–76^. Above 1:1 equiline of (${Ca}^{2+}+ {Mg}^{2+}$) and ${HCO}_{3}^{-}$ suggests the predominance of ${Ca}^{2+}$ and ${Mg}^{2+}$ over ${HCO}_{3}^{-}$ due to rock weathering or cation exchange, whereas below it verifies feldspar minerals containing carbonic acid are responsible for the release of ${HCO}_{3}^{-}$ into the groundwater (Eq. 9)^77,78^. In this study, all chemical data plotted significantly above the 1:1 line of (${Ca}^{2+}+ {Mg}^{2+}$) and ${HCO}_{3}^{-}$ (Fig. 6B), indicating the predominance of rock weathering and ion exchange processes. This pattern suggests additional sources of alkaline earth metals, likely from silicate weathering. The weak correlation further implies that carbonate weathering is not the dominant process governing groundwater chemistry. The inverse to weak relationship between (SO_4_^2–^+ Cl^–^ and HCO_3_^–^ indicates distinct geochemical controls on these ions (Fig. 6C). Elevated SO_4_^2–^ + Cl^–^ relative to HCO_3_^–^ in these samples suggest contributions from anthropogenic sources such as agricultural inputs, rather than carbonic acid-driven weathering alone. In general, the groundwater system receives an equal amount of these ions from the dissolution of ${Ca}^{2+}$ and ${Mg}^{2+}$ silicates and ${HCO}_{3}^{-}$ and ${SO}_{4}^{2-}$ linked with the soils^79^, (Fig. 6D). The reverse-ion exchange mediated by the generation of (${Ca}^{2+}+ {Mg}^{2+}$) ions from the aquifer material is shown by the high (${Ca}^{2+}+ {Mg}^{2+}$) compared to (${HCO}_{3}^{-}$ +${SO}_{4}^{2-}$). The removal of ${Ca}^{2+}$ and ${Mg}^{2+}$ ions from the water by the cation exchange process is indicated by excess ${HCO}_{3}^{-}$ derived from the weathering of ${Na}^{+}$ and ${K}^{+}$ silicates^80^. Most samples cluster above the equiline, indicating that ${Ca}^{2+}+ {Mg}^{2+}$ exceed the combined contribution from bicarbonate and sulfate. This imbalance is characteristic of ion exchange processes, particularly reverse cation exchange, where Na^+^ in groundwater is exchanged for ${Ca}^{2+}$ and ${Mg}^{2+}$ from aquifer materials. The scatter further supports the influence of multiple concurrent processes rather than a single dominant reaction. Furthermore, we observed the plots of (${Ca}^{2+}+ {Mg}^{2+}$) *vs.* (${Na}^{+}+ {K}^{+}$) appear almost evenly distributed between the ions in the groundwater (Fig. 6E), with the spread of data points indicating spatial variability in water–rock interaction intensity and exchange reactions. This pattern signifies that groundwater chemistry is largely controlled by early-stage water–rock interaction, where mineral weathering release Ca and Mg into solution before extensive alkali enrichment occurs. The subordinate ${Na}^{+}+ {K}^{+}$ contents suggest that prolonged residence time and ion exchange, which typically shift waters toward Na-rich facies, are limited. The observed scatter reflects spatial heterogeneity in lithology and exchange capacity of aquifer materials, implying variable intensities of silicate weathering and localized ion exchange. In the (${Ca}^{2+}+ {Mg}^{2+}$) *vs.* total cations (${Ca}^{2+}+ {Mg}^{2+}+{Na}^{+}+{K}^{+}$) diagram of the samples, all chemical points diverge from the equiline and shift their trend in the direction of the total cations (Fig. 6F), demonstrating that alkaline earth metals constitute a major proportion of total cations in the groundwater. This is the outcome of the cations’ overall contribution from the source. This dominance confirms that mineral weathering, particularly of Ca- and Mg-bearing silicates, is the principal source of dissolved ions. The limited deviation from the equiline suggests that while alkali metals (Na⁺ + K⁺) contribute to total cations, their role is secondary. This relationship is characteristic of relatively fresh recharge waters that have undergone moderate geochemical evolution without extensive ion exchange or evaporative concentration. As a result, these diagrams make it evident that silicate weathering is the primary mechanism governing water chemistry in the study area, with significant modification by cation exchange processes. Localized anthropogenic inputs likely contribute to ${SO}_{4}^{2-}$ and ${Cl}^{-}$ enrichment in some samples. The predominance of Ca–Mg over Na–K suggests a relatively early to intermediate stage of hydrogeochemical evolution, consistent with active recharge and limited residence time within the aquifer system. The observed ionic relationships are consistent with groundwater-rock interaction within a Basement Complex terrain dominated by migmatite gneiss and granitic lithologies. Weathering of primary silicate minerals (e.g., feldspars, biotite and hornblende) contributes major cations (Ca^2+^, Mg^2+^, Na^+^) to groundwater, while secondary clay minerals formed during weathering provide active surfaces for ion-exchange reactions. Thus, the hydrochemical evolution reflects the coupled influence of silicate rock weathering and ion exchange processes.

#### Hydrochemical evolution mechanism

**Fig. 7:**
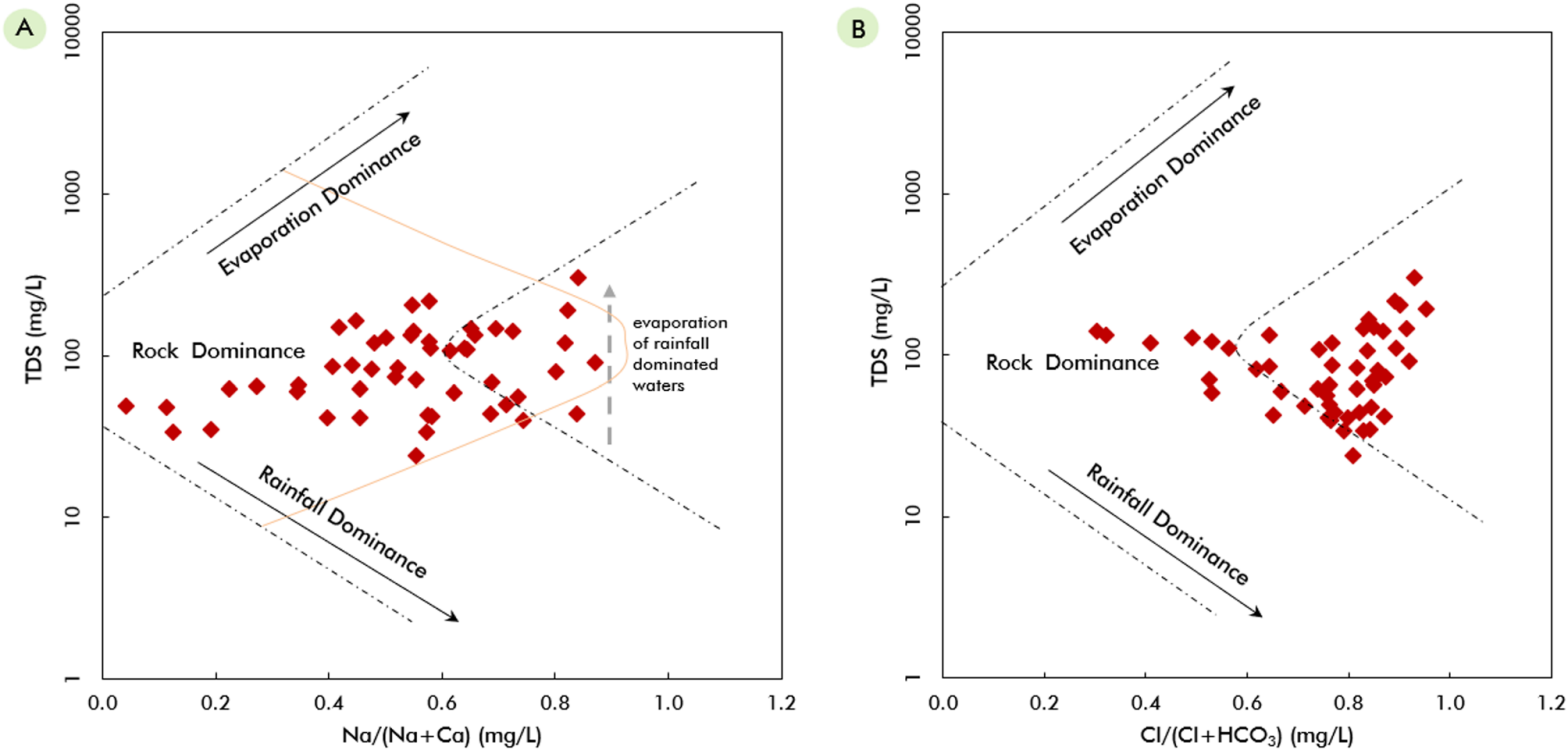
Gibbs diagrams illustrating the mechanisms governing the major ion chemistry of the studied groundwater samples from the Oye-Ekiti area: (**a**) cations: TDS vs. Na/(Na + Ca); and (**b**) anions: TDS vs. Cl/(Cl + HCO_3_).

**Fig. 8:**
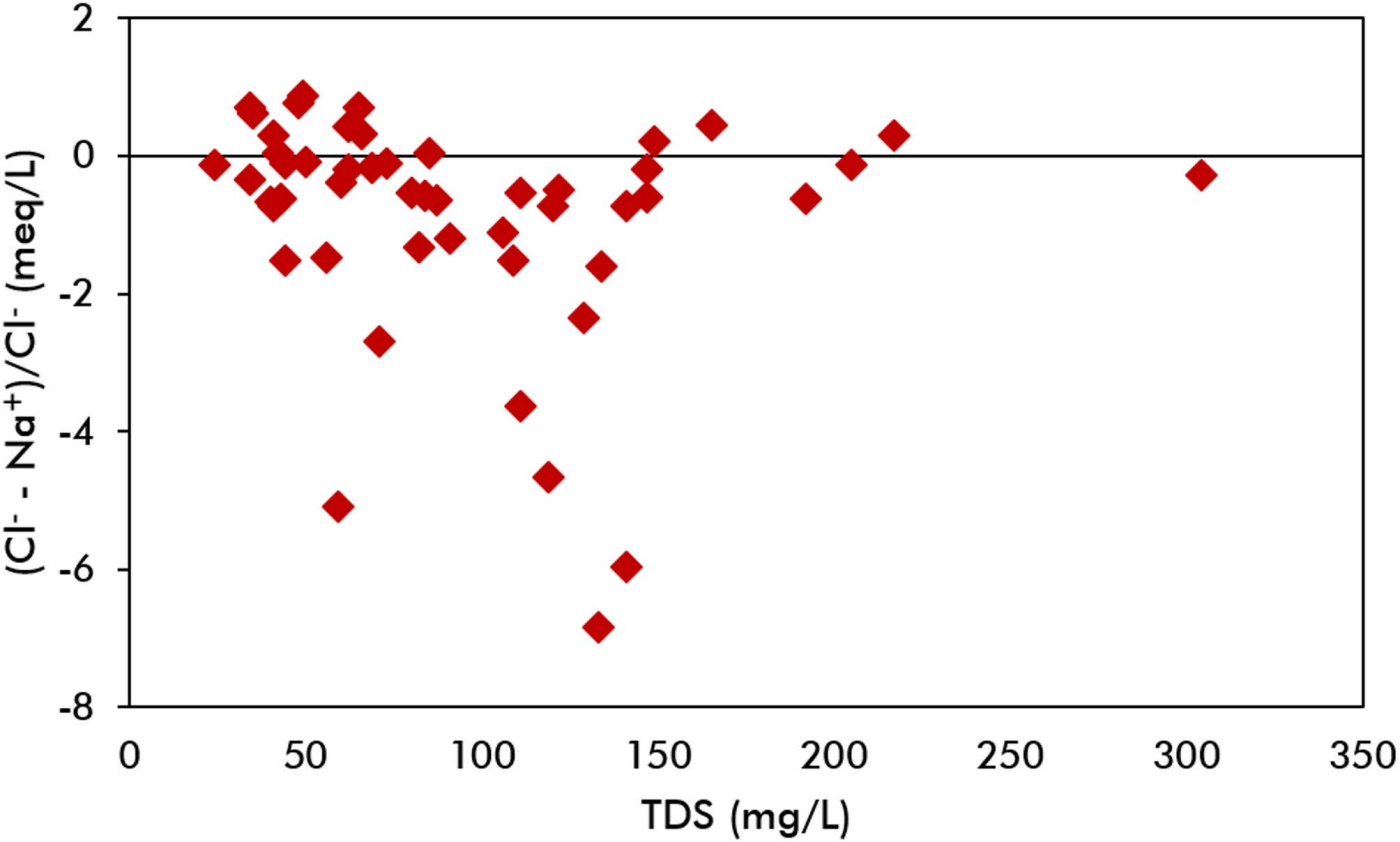
Bivariate relationship between (Cl–Na)/Cl and TDS of the studied groundwater samples from the Oye-Ekiti area.

Understanding how groundwater interacts with the minerals found within the aquifer is crucial to understanding its chemistry. It was observed that the groundwater samples from Oye-Ekiti plotted within the rock-dominance field, reflecting the significance of rock-water interactions as the primary source of dissolved ions (Fig. 7). Gibbs diagrams also indicate that none of the groundwater samples plot within the rainfall dominance field, while a few samples extending into the evaporation dominance field, suggesting localized concentration of dissolved ions due to evaporative processes. Chemical ion ratios can be used to identify the impact of hydrochemical processes affecting water quality, such as ion-exchange, mixing, and leaching. In order to comprehend the connections between the ions and variables influencing groundwater chemistry, this study used the interactions between various ionic concentrations. The majority of the groundwater samples (~ 98%) exhibited higher Na^+^ than Ca^2+^, Mg^2+^, and HCO_3_^−^, which may be explained by silicate weathering, according to the ratios of Ca^2+^/Na^+^ versus (vs.) HCO_3_^−^/Na^+^ (Fig. 4). Cation exchange process between Na^+^ and K^+^ dissolved in groundwater with Ca^2+^ and Mg^2+^ entrenched in the aquifer matrix has been demonstrated by the fact that 26% of the water samples in this study showed a positive value for the relation of (Cl^−^−Na^+^)/Cl^−^ *vs.* TDS (Fig. 8). Conversely, negative values found in 74% of groundwater samples suggest a reverse-ion exchange process. The bivariate ionic relationships and molar ratios in this study indicate that silicate mineral weathering is a primary control on major cation release in groundwater, with secondary cation exchange altering Na⁺, Ca^2+^, and Mg^2^⁺ proportions. The geochemical evolution of groundwater in the study area reflects the influence of water–rock interaction and secondary ion exchange processes rather than rainfall dominance. The geology of Oye-Ekiti is typical of the Precambrian Basement Complex terrains comprising granitoids (including biotite and porphyritic granites), gneiss, schist, and associated siliceous rocks rich in feldspar (plagioclase, K‑feldspar) and ferromagnesian minerals such as biotite and hornblende, which are common lithologies in southwestern Nigerian basement rocks^10,38,39^. Similar hydrochemical investigations in basement aquifers across Nigeria have attributed groundwater chemical evolution to silicate weathering and rock-water interaction, with ion exchange modifying ionic composition in the saprolite and fractured zones (e.g., Na^+^/Cl⁻ and Ca^2+^ + Mg^2+^/HCO₃^–^ trends)^81–83^. These geochemical processes, rather than exclusively meteoric influences, govern the hydrochemical signatures observed in the study area.

#### Ion exchange

Ion-exchange mechanisms are crucial for understanding groundwater chemistry because they provide insights into the composition and interactions with the rock units^84^.

**Fig. 9:**
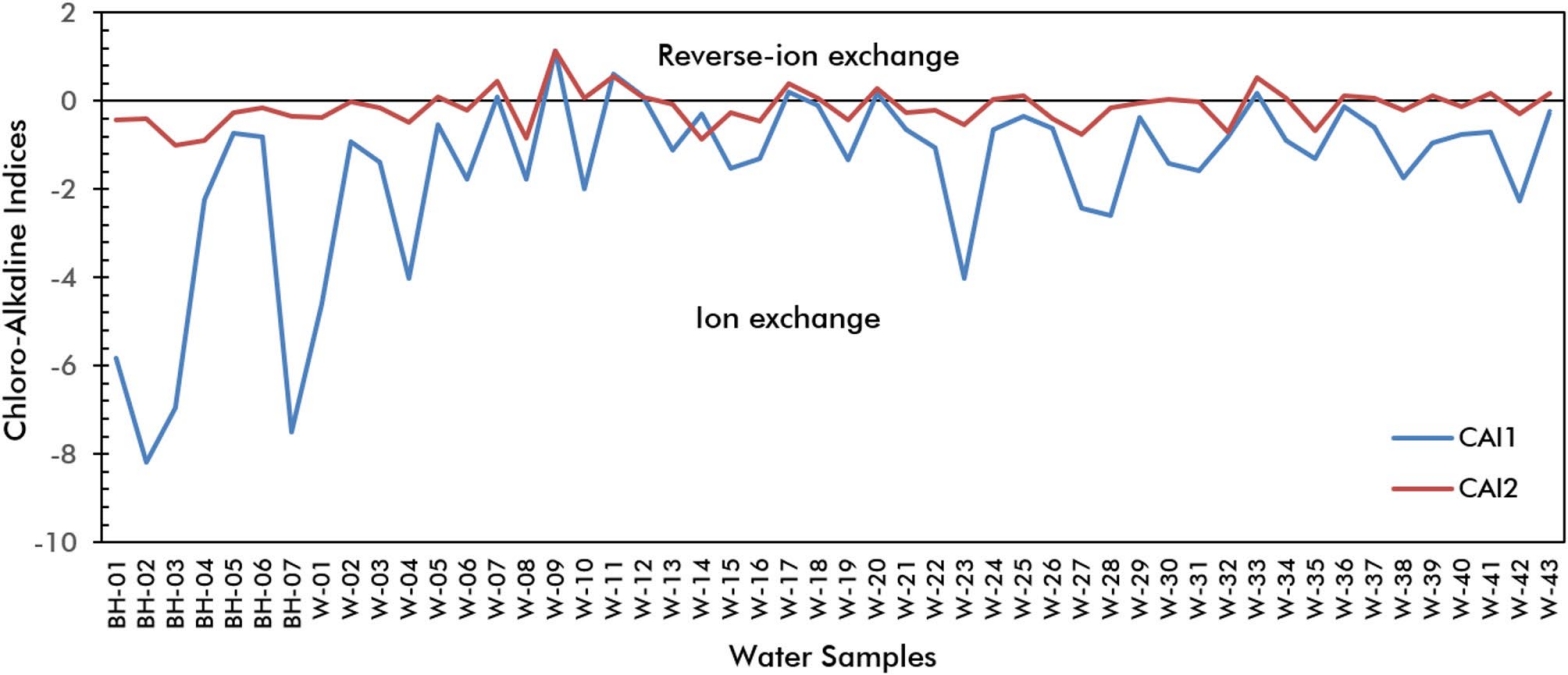
Schoeller’s ion exchange reactions (Chloro-Alkaline Indices—CAI_1_ and CAI_2_) of the studied groundwater samples from the Oye-Ekiti area.

Among the analyzed samples, seven exhibited positive CAI_1_ values and 19 samples had positive CAI_2_ values. The average CAI_1_ and CAI_2_ values are − 1.57 and − 0.15, respectively. The majority of water samples in the study area display negative CAI_1_ and CAI_2_ indices (Fig. 9). The CAI results indicate that ion-exchange is the main hydrochemical process governing the groundwater chemistry in the area, despite the observation of both ion-exchange and reverse-ion exchange instances (Fig. 9). For ion-exchange and reverse-ion exchange processes, respectively, the probable reactions (Eqs. 11 and 12) can be expressed as follows^40,44^:

11 $$2Na+\left({{Ca}_{1-x}Mg}_{x}\right) -X\to \left(1-x\right)Ca+xMg +{Na}_{2}-X (0\le x\ge 1)$$

12 $$\left(1-x\right)Ca + xMg + {Na}_{2}-X\to \left({Ca}_{1-x}{Mg}_{x}\right)-X+ 2Na (0\le x\le 1)$$

where X indicates the exchanging solid surface.

#### Hydrogeochemical modeling

Table 2 summarizes the mineralogical phases calculated from the overall dataset of examined physicochemical parameters in the Oye-Ekiti area, providing information on the calculated Saturation Indices (SIs) for the 50 groundwater samples. All mineral phases were identified as undersaturated, attributed to the relatively low alkalinity of the aqueous solution, which suggests ongoing dissolution within the aquifer matrix. The in-depth study of water–rock interactions and the dissolution of mineralogical phases reveals an increase in major ions in groundwater resources in the study area. The results of geochemical modeling support the findings derived from statistical analysis and hydrogeochemical diagrams (e.g., Gibbs plots, bivariate diagrams, etc*.*), reinforcing the hypothesis that water–rock interactions and geochemical reactions influence groundwater chemistry.

**Table 2:** Descriptive statistics (Min. = Minimum value, Max. = Maximum value, Mean = average value, Median = Median value) of calculated Saturation Indices (SI) of selected mineral phases and Chloro-Alkaline Indices (CAI, meq/L) for groundwater samples (N = 50) from the Oye-Ekiti area.

Mineral phase	Min	Max	Mean	Median
SI_Anhydrite_	− 3.58	− 2.01	− 2.79	− 2.77
SI_Aragonite_	− 3.79	− 1.01	− 2.49	− 2.58
SI_Artinite_	− 16.3	− 8.27	− 12.68	− 12.83
SI_Brucite_	− 10.35	− 5.56	− 8.52	− 8.64
SI_Epsomite_	− 7.25	− 4.73	− 5.85	− 5.81
SI_Gypsum_	− 3.61	− 2.05	− 2.84	− 2.81
SI_Halite_	− 9.45	− 6.73	− 8.03	− 8.04
SI_Huntite_	− 20.74	− 7.66	− 14.85	− 15.11
SI_Hydromagnesite_	− 37.33	− 18.19	− 28.31	− 28.63
SI_Magnesite_	− 5.11	− 1.41	− 3.31	− 3.38
SI_Mirabilite_	− 11.68	− 7.36	− 9.16	− 9.12
SI_Natron_	− 14.56	− 10.75	− 12.34	− 12.3
SI_Nesquehonite_	− 7.52	− 3.82	− 5.72	− 5.79
SI_Thenardite_	− 12.53	− 8.19	− 10.02	− 9.95
SI_Thermonatrite_	− 15.92	− 12.06	− 13.7	− 13.68
SI_Dolomite_	− 7.45	− 1.79	− 5.16	− 5.19
SI_Calcite_	− 3.65	− 0.87	− 2.35	− 2.44
CAI_1_	− 7.34	0.62	− 1.22	− 0.76
CAI_2_	− 0.83	0.27	− 0.2	− 0.22

### Factors controlling groundwater chemistry

Correlation analysis, FA, and R-/Q modes HCA collectively provide insight into the dominant processes controlling groundwater chemistry in the study area. The observed relationships are discussed here in terms of geogenic (mineral dissolution and weathering) and anthropogenic controls.

#### Geogenic processes: mineral dissolution and weathering

**Fig. 10:**
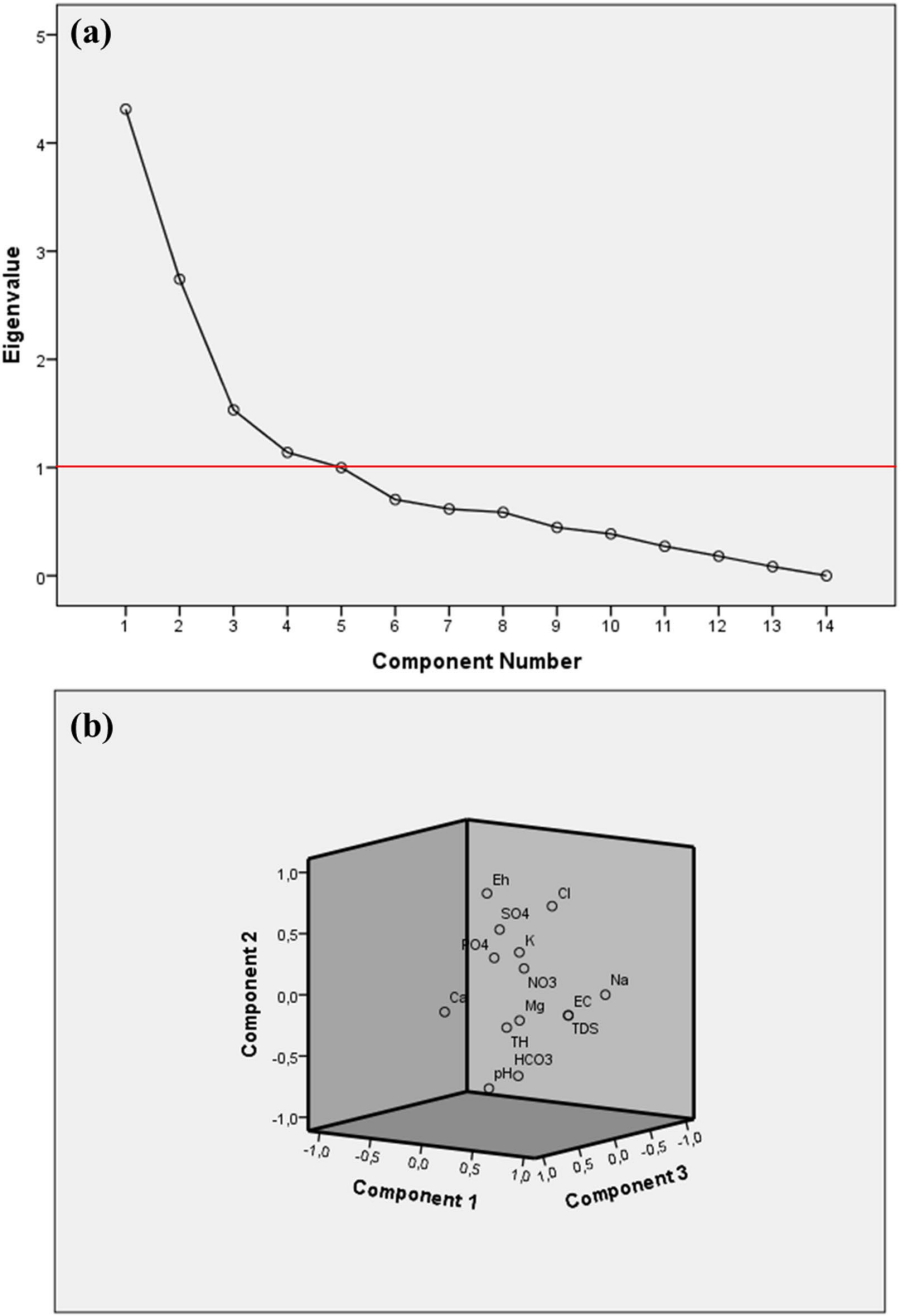
(**a**) Scree plot of the eigenvalues for the 50 groundwater samples from the Oye-Ekiti area. (**b**) The loading plot of factor scores (components in rotated space) for the 50 groundwater samples from the Oye-Ekiti area.

The Spearman’s rank correlation analysis, FA, and HCA were conducted on 50 groundwater samples and 14 parameters from the Oye-Ekiti area to identify the dominant natural processes influencing groundwater chemistry. The Spearman’s rank correlation matrix (Table 3) demonstrates strong to very strong statistically significant correlations (*p* < 0.05) among parameters related to groundwater mineralization. Notable relationships include, the most remarkable (i.e., from moderate to very strong) statistically significant (i.e., *p*-value < 0.05 level) correlation coefficients are as follows: TH- Mg^2+^ (ρ = 0.922), EC-TH (ρ = 0.657), EC-Mg^2+^ (ρ = 0.652), EC-Na^+^ (ρ = 0.658), TDS-TH (ρ = 0.656), TDS-Mg^2+^ (ρ = 0.651), and TDS-Na^+^ (ρ = 0.660). Moderate correlations are also observed between EC-HCO_3_^-^ (ρ = 0.428), TDS-HCO_3_^-^ (ρ = 0.427), EC-Cl^−^ (ρ = 0.425), and TDS-Cl^−^ (ρ = 0.432). These associations indicate that groundwater chemistry is largely governed by mineral dissolution and water–rock interaction. This interpretation is reinforced by FA results. A total of four factors with eigenvalues greater than 1 explain 69.5% of the total variance. The dataset is statistically suitable for FA, as indicated by a KMO value of 0.671 and a Bartlett’s test significance of 0.000. The scree plot (Fig. 10a) illustrated that four (4) components have eigenvalues > 1 according to Kaiser’s criterion^58^, explaining 71.06% of the total variance (Table 4). The results were statistically significant, as indicated by the KMO coefficient > 0.5 (actual value = 0.671), and the data were valid and appropriate for FA, as indicated by Bartlett’s test of sphericity (*p*= 0.000^85^;). The factor plot in the rotated space of FA is illustrated in Fig. 10b.

**Table 3:** Spearman’s rank correlation matrix and corresponding *p* values for a dataset comprising 14 parameters across 50 groundwater samples from the Oye-Ekiti area.

Parameter	pH	Eh	EC	TH	Ca	Mg	Na	K	HCO_3_	Cl	SO_4_	NO_3_	PO_4_	TDS
pH	1.000													
Eh	− 0.435**	1.000												
EC	0.363**	0.000	1.000											
TH	0.384**	− 0.100	0.657**	1.000										
Ca	0.280*	− 0.010	0.301*	0.305*	1.000									
Mg	0.390**	− 0.060	0.652**	0.922**	0.230	1.000								
Na	0.020	− 0.050	0.658**	0.371**	0.020	0.320*	1.000							
K	− 0.060	0.160	0.180	0.080	− 0.150	0.160	0.110	1.000						
HCO_3_	0.318*	− 0.160	0.428**	0.280	0.100	0.288*	0.314*	− 0.120	1.000					
Cl	− 0.382**	0.342*	0.425**	0.170	− 0.020	0.250	0.443**	0.296*	− 0.010	1.000				
SO_4_	− 0.120	0.250	0.200	0.120	0.110	0.230	0.170	0.497**	− 0.150	0.290*	1.000			
NO_3_	− 0.040	0.140	0.297*	0.030	0.140	0.020	0.285*	0.130	0.130	0.326*	0.120	1.000		
PO_4_	− 0.030	0.010	− 0.070	0.080	0.100	0.070	− 0.030	− 0.190	− 0.030	− 0.120	− 0.210	− 0.060	1.000	
TDS	0.363**	0.003	1.000**	0.656**	0.297*	0.651**	0.660**	0.180	0.427**	0.432**	0.200	0.299*	− 0.080	1.000

**Correlation is significant at the 0.01 level (2-tailed).

*Correlation is significant at the 0.05 level (2-tailed).

**Table 4:** Varimax rotated principal components of 50 groundwater samples from the Oye-Ekiti area.

Parameter	Component			
	F1	F2	F3	F4
TDS	0.884	− 0.079	0.321	0.156
EC	0.883	− 0.081	0.319	0.157
Na	0.846	0.002	− 0.251	− 0.020
Mg	0.514	− 0.137	0.476	0.460
NO_3_	0.345	0.227	0.172	− 0.297
Eh	− 0.117	0.773	0.029	− 0.076
pH	0.092	− 0.760	0.300	0.194
Cl	0.503	0.728	0.004	0.056
HCO_3_	0.370	− 0.631	0.288	− 0.169
SO_4_	0.142	0.532	0.224	0.487
Ca	0.038	− 0.062	0.843	− 0.196
TH	0.475	− 0.181	0.600	0.351
K	0.210	0.327	0.045	0.721
PO_4_	0.034	0.278	0.144	− 0.697
Initial eigenvalues of variances in %	30.8	19.6	10.9	8.1
Cumulative % of variance	30.8	50.4	61.3	69.5

**Fig. 11:**
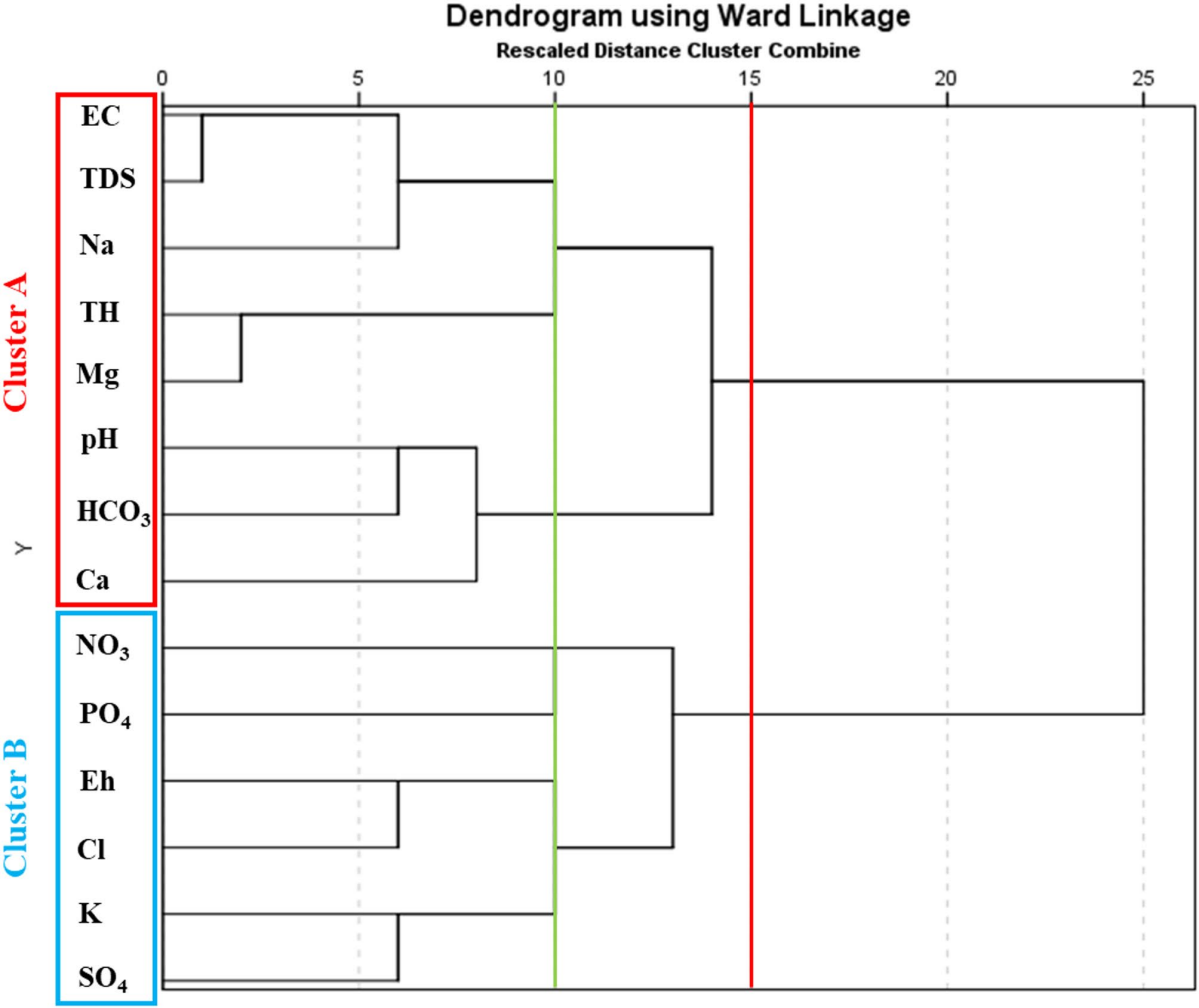
Dendrogram in R-mode HCA for 15 parameters determined in groundwater samples from a total of 50 water samples from the Oye-Ekiti area. The red and green lines represent the linkage distance used to create different distinct clusters.

**Fig. 12:**
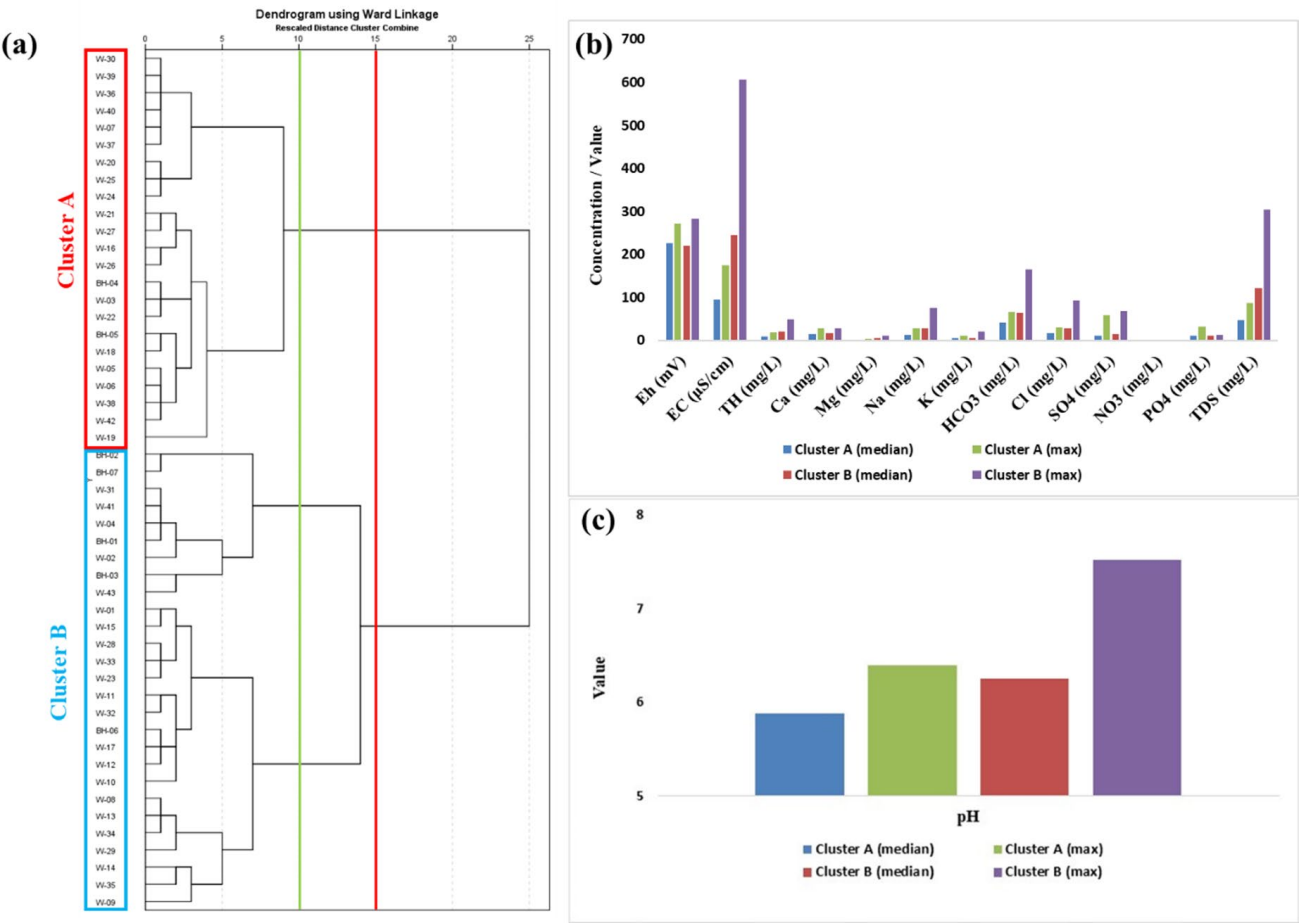
(**a**) Dendrogram in Q-mode HCA all groundwater samples from the Oye-Ekiti area. The red and green lines represent the linkage distance used to create different distinct clusters. (**b**, **c**) Median and maximum values of the examined parameters among Clusters A and B of the Q-mode HCA from the Oye-Ekiti area.

The first factor (FA1) accounts for 30.8% of the total variance and shows strong positive loadings for TDS (0.884), EC (0.883), and Na^+^ (0.846), moderate positive loadings for Mg^2+^ (0.514) and Cl^−^ (0.503), and weak positive loadings for TH (0.475), HCO_3_^−^ (0.37), and NO_3_^−^ (0.345). This factor represents the dissolved ion load in the groundwater of the Oye-Ekiti area and it is associated with TDS and the most major ions, indicating the dissolution of various mineral phases within the aquifer. TDS and EC reflect the overall ionic content of groundwater controlled by dissolved salts derived from aquifer materials^86^. Chloride mobilization is primarily linked to rock weathering processes (Fernández-Turiel et al.^93^). The second factor (FA2) accounted for 19.6% of the total variance, strong positive loadings for Eh (0.773), negative loadings for pH (− 0.76), moderate positive loadings for Cl^−^ (0.728) and SO_4_^2−^ (0.534), moderate negative loading for HCO_3_^−^ (− 0.631), and weak positive loading for K^+^ (0.327). This factor reflects groundwater alkalinity and buffering capacity, which develops as recharge water interacts with soil and rock materials. Alkalinity is controlled by silicate weathering and carbonate mineral dissolution, particularly calcite, through HCO_3_^−^ hydrolysis^87–89^. R- and Q- modes HCA were performed to classify hydrochemical parameters and sampled sites, as well as to reveal underlying relationships among these parameters and samples within the Oye-Ekiti area (Figs. 11 and 12). Parameters (for R-mode or sample sites (for Q-mode with a linkage distance of less than 10 (green line; Fig. 11) and around 15 (red line; Fig. 11) were grouped into clusters to share similar hydrogeochemical patterns. The dendrograms (Figs. 11 and 12) demonstrate that, at higher linkage distances, the parameters form two main clusters, while the sample sites are also grouped into two clusters. The green line in the dendrograms highlights hydrogeochemical similarities between parameters and samples at lower linkage distances, suggesting closer relationships. R-mode HCA further supports these interpretations. Two distinct categories, designated Clusters A and B, are visible when an imaginary red vertical line is placed across the dendrogram at a linkage distance of 15 (Fig. 11). Cluster A is thought to be the outcome of all ions dissolving completely, providing information on the quality of the groundwater. Cluster A is thus linked to FA1 and reflects geogenic mineralization. TDS values range from 24 to 304 mg/L, with a median of 81 mg/L (Table 1), indicating variable but generally low mineralization controlled by aquifer lithology. This cluster is dominated by Na^+^, Mg^2+^, EC, TH, and TDS, pH, HCO_3_^-^, and Ca^2+^, suggesting a bedrock-derived source of solutes. Furthermore, the third factor (FA3) accounts for 10.9% of the total variance, with a strong positive loading for Ca^2+^ (0.843), a moderate positive loading for TH (0.6), weak positive loadings for TDS (0.321), EC (0.319), Mg^2+^ (0.419), and pH (0.3), highlighting the relationship between pH-Ca^2+^-HCO_3_^-^ that is revealed in Cluster A of R-mode HCA (see green line), indicating the buffering capacity of the water. Alkalinity also shows a statistical relationship with Ca^2+^, attributed to the presence of carbonate minerals, primarily CaCO_3_. Furthermore, when CO_2_ dissolves in an aqueous solution, CO_2_ (aq) readily reacts to form carbonic acid (H_2_CO_3_), which dissolves carbonate and silicate minerals^53,89^.

#### Anthropogenic influences

Anthropogenic effects are primarily discerned through correlations between nitrate and phosphate behavior and through FA and HCA. Spearman’s correlation reveals moderate associations between Na⁺–Cl^−^ (ρ = 0.443) and K⁺–SO₄^2^⁻ (ρ = 0.497), suggesting inputs beyond purely geogenic sources. A notable negative correlation between pH and Eh (ρ = − 0.435) indicates the influence of redox-sensitive processes on certain contaminants.

The fourth factor (FA4) explained 8.1% of the total variance, with a moderate positive loading for K^+^ (0.721), moderate negative loading for PO_4_^3−^ (− 0.697), weak positive loading for SO_4_^2−^ (0.487), Mg^2+^ (0.46), and TH (0.351). These factors reflect anthropogenic inputs associated with agricultural practices, sewage effluents, manure application, and waste disposal.

The co-loading of certain dissolved ions and PO_4_^3−^ in FA4 suggests contributions from agrochemical products or fertilizer-derived minerals, such as apatite-group minerals (e.g., fluorapatite, chlorapatite, hydroxylapatite, carbonate-rich apatite, etc*.*), including superphosphate and triple superphosphate^90^. Although NO_3_^−^ concentrations are low (up to 0.91 mg/L), PO_4_^3-^ concentrations are elevated (up to 32.52 mg/L). The limited variance explained by FA4 implies that anthropogenic impacts are localized rather than widespread across the area.

R-mode HCA Cluster B includes K^+^, SO_4_^2−^, Eh, PO_4_^3−^, Cl^−^, and NO_3_^−^, further confirming anthropogenic influence. Its proximity to Cluster A suggests that some ions, particularly K^+^ and SO_4_^2^, originate from both natural weathering and human activities. The behaviour of nitrate and phosphate is also linked to redox conditions, particularly Eh, as reported in a previous study^91^.

It is noted that the results of the R-mode HCA are supported by Spearman’s correlation coefficients and FA approaches. Considering the Q-mode HCA clusters, an imaginary red vertical line drawn across the dendrogram at a linkage distance of 15 separates two distinct clusters labeled as Clusters A and B (Fig. 12), distinguished by their dominant chemical compositions. Cluster B exhibits higher concentrations of most dissolved ions (e.g., Na^+^, Cl^−^, Ca^2+^, Mg^2+^) as well as elevated levels of TDS, EC, and alkalinity compared to Cluster A. Significant differences in chemical composition between the two clusters are evident from their median and maximum values (Fig. 12b, c). Generally, groundwater chemistry in the study area is controlled predominantly by natural geological processes, with localized anthropogenic modification superimposed on the geogenic baseline.

### Stable isotopic composition

**Fig. 13:**
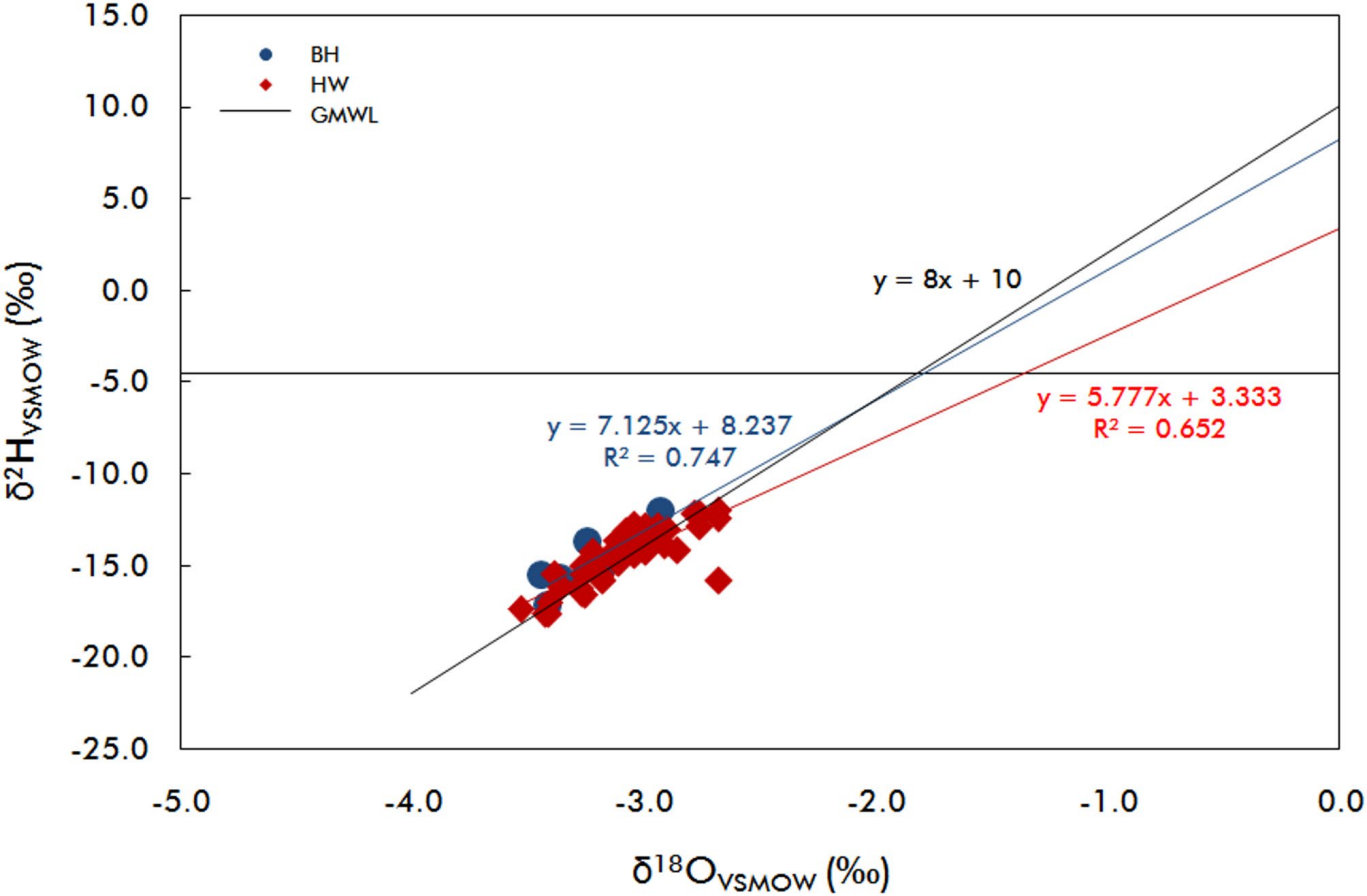
Bivariate relationship between δ^2^H and δ^18^O ratios of the studied groundwater samples from the Oye-Ekiti area.

Table 1 summarizes the isotopes analysis results for the groundwater samples that have been collected from the study area. Isotopic composition ranges from − 3.53 to − 2.68‰, with a mean value of − 3.11‰ for δ^18^O (median = − 3.11‰), and the δ^2^H ranges between − 17.65 and − 11.96‰, with a mean value of − 14.53‰ (median = − 14.30‰). A summary of the isotope data (Table 5) and meteoric lines (slope and intercept, Table 6) for this study and other locations in SW Nigeria are provided for a clear numeric comparison. Table 6 shows MWL slope, intercept, and standard errors. The isotopic composition of groundwater from both borehole and hand-dug well samples show similar characteristics, closely following the GMWL and the regional LMWL for the Niger Delta (δ^2^H = 7.7 × δ^18^O + 10.2^29^;). This similarity underscores the significant contribution of local rainfall in recharging the groundwater in the area. The narrow range of δ^18^O and δ^2^H values also suggests a relatively homogenous and well-mixed groundwater system (Fig. 13). However, some groundwater samples plot to the right of the GMWL, suggesting possible evaporative enrichment of the meteoric water during rainfall or recharge through the vadose zone. Borehole samples yield a groundwater δ^2^H–δ^1^⁸O regression line of δ^2^H = 7.13 × δ^18^O + 8.23, r^2^ = 0.75, (n = 7) and generally lie above both the GMWL and regional LMWL, suggesting minimal post-recharge evaporation. Hand-dug well samples, however, show limited to modest evaporative enrichment, with groundwater δ^2^H–δ^1^⁸O regression line of δ^2^H = 6.66 × δ^18^O + 6.1, r^2^ = 0.80 (n = 41; Fig. 13). Similarly, most d-excess values (8.7–12.2‰) cluster near the global average of 10‰, further suggesting minimal isotopic fractionation. Additionally, the lower δ^18^O values and d-excess values near 10‰ suggest that recharge likely occurred from precipitation with minimal evaporation before infiltration, suggesting direct or rapid percolation through the unsaturated zone under humid or moderate climatic conditions. This is further enhanced by high secondary porosity and permeability controlled by the local geology^92^. Notably, the lowest d-excess value of W-19 (5.6‰) may be attributed to a relatively higher degree of evaporation before or during groundwater recharge.

**Table 5:** Summary of groundwater δ^18^O, δ^2^H, d-excess data and regression line slope and intercept.

Station	n	δ^18^O_VSMOW_ (‰)			δ^2^H_VSMOW_ (‰)			*d-excess*_VSMOW_ (‰)		
		mean	max	min	mean	max	min	mean	max	min
This study	48	− 3.1 ± 0.2	− 2.7	− 10.9	− 14.5 ± 1.6	− 11.6	− 17.7	10.4 ± 0.8	12.2	8.7
Ekiti, SW Nigeria*	27	− 3.0 ± 0.4	− 2.6	− 4.2	− 14.9 ± 2.7	− 10.2	− 22.3	9.45 ± 0.9	11.0	6.8
Lagos Coastal basin, SW Nigeria^†^	29	− 2.51 ± 1.1	− 0.1	− 4.8	− 11.6 ± 5.6	− 0.9	− 24.8	6.4 ± 5.7	13.9	− 2.3

*The Ekiti, SW Nigeria data is reported byTalabi and Tijani^10^.

^†^The Lagos Coastal basin data is reported byYusuf et al.^30^.

**Table 6:** Meteoric Water Lines for Nigeria: regressions of precipitation δ^18^O and δ^2^H values.

Location	n	R^2^	Slope	Intercept	Sample type
This study	7	0.75	7.13 ± 1.8	8.23 ± 5.9	Groundwater/borehole
This study	41	0.80	6.66 ± 0.5	6.15 ± 1.6	Groundwater/hand-dug well
Ekiti, SW Nigeria*	27	0.94	6.50 ± 0.3	4.89 ± 1.0	Groundwater
Lagos Coastal basin, SW Nigeria^†^	29	0.49	3.48 ± 0.7	− 2.79 ± 1.8	Groundwater
Niger Delta, Nigeria**	–	0.87	7.70	10.2	Precipitation

*The Ekiti, SW Nigeria data is reported by Talabi and Tijani^10^.

^†^The Lagos Coastal basin data is reported by Yusuf et al.^30^.

**The The Niger Delta precipitation data is reported by Ohwoghere-Asuma et al.^29^.

**Fig. 14:**
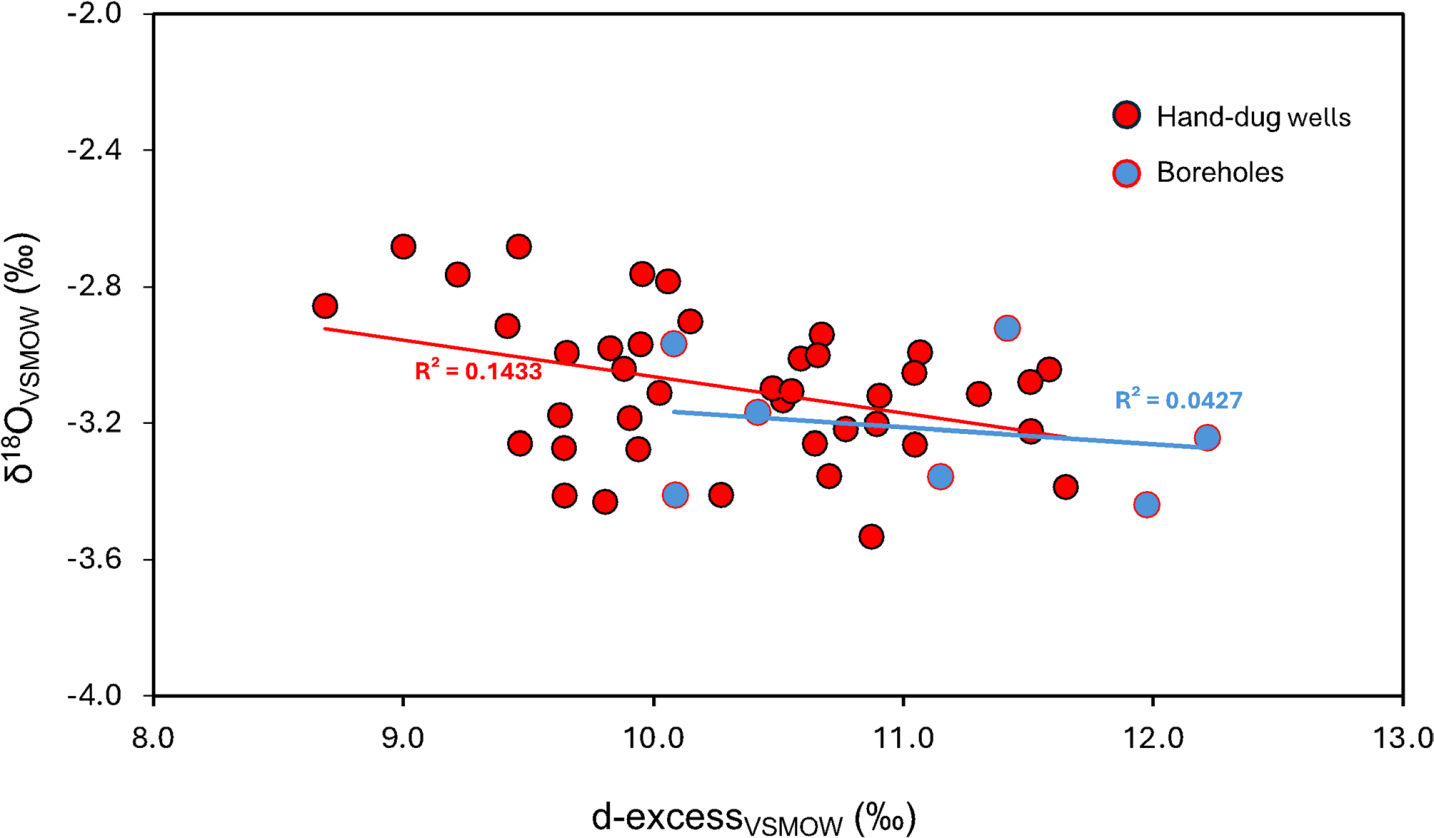
A plot of d-excess versus δ^18^O for hand-dug well and borehole samples of the groundwater samples from the Oye-Ekiti area (W-19 is excluded from the regression).

Evaporated groundwater typically displays a low d-excess value and exhibits a negative correlation between δ^18^O and d-excess. Figure 14 illustrates a weak correlation between δ^18^O and d-excess (R^2^ = 0.14) for hand-dug well and (R^2^ = 0.04) for borehole samples, suggesting that only a small portion of the d-excess variability is associated with δ^18^O enrichment. The low correlation, together with evidence of limited evaporative enrichment, indicates that d-excess variability is likely controlled by multiple processes, including variability in precipitation sources, seasonal recharge signals, and aquifer mixing. Further evaluation of seasonal recharge dynamics is needed to better constrain these influences.

### Limitations specific to dry-season sampling

This study is based exclusively on groundwater samples collected during the dry season (January–February 2022), and therefore the hydrochemical and isotopic results represent conditions typical of this period. Under dry-season conditions, enhanced evapotranspiration and reduced recharge may lead to increased solute concentrations in shallow groundwater, which means that evaporative concentration in hand-dug wells may be somewhat overestimated relative to wetter periods. Furthermore, recharge dynamics associated with the wet season, when intense rainfall between April and October can significantly influence groundwater dilution and flow pathways, were not captured in the present dataset. The δ^18^O–δ^2^H signatures measured in this study therefore reflect groundwater recharged by rainfall occurring during the months preceding sampling, potentially missing seasonal isotopic extremes associated with peak monsoon precipitation.

In addition, interpretation of isotopic evaporation effects should be treated with caution due to the limited borehole sample size and the moderate difference between the hand-dug well regression slope and the regional meteoric water line. Although the observed isotope relationships suggest predominantly meteoric recharge with only modest evaporative influence, quantitative assessment of evaporation losses would require additional approaches such as vadose-zone evaporation modelling, higher-frequency seasonal sampling, or comparison with time-resolved rainfall isotope datasets. These analyses were beyond the scope of the present study but represent important directions for future work.

### Assessment of proposed hypotheses

The formulated hypotheses (H_1_–H_3_) were evaluated using isotopic, hydrochemical, and modelling results. The δ^2^H–δ^18^O relationship closely matched the regional LMWL (δ^2^H = 7.7 × δ^18^O + 10.2, r^2^ = 0.87), confirming H_1_ that groundwater recharge originates from local precipitation with minimal pre-infiltration evaporation. The dominance of Ca–Mg–HCO_3_ facies, rock-dominance field position in the Gibbs plots, and negative carbonate saturation indices (SI_calcite < 0) support H_2_, demonstrating that silicate weathering is the principal geochemical control. Isotopic enrichment and slightly higher TDS values in hand-dug wells relative to boreholes support H_3_, indicating shallow evaporation and depth-dependent fractionation effects. Collectively, these findings demonstrate the coherence among field hydrochemistry, isotopic evidence, mineral equilibria, and validate the proposed conceptual model of recharge and geochemical evolution in Oye-Ekiti groundwater.

## Conclusions

This study provides the first city-scale, dry-season integrated hydrochemical, isotopic (δ^18^O–δ^2^H), multivariate statistical, and geochemical-modelling assessment of groundwater in the crystalline basement aquifer system of Oye-Ekiti, southwestern Nigeria. The combined use of these techniques demonstrate that groundwater chemistry is predominantly controlled by natural geogenic processes, with secondary modification by localized anthropogenic inputs, and yielded the following main findings:

1. Groundwater is generally fresh, soft, and low-salinity, with TDS within the WHO and NIS drinking-water guide values. However, about 88% of samples exhibit slightly acidic conditions (pH < 6.5), reflecting the low buffering capacity of silicate-dominated migmatite–gneiss and granitic lithologies. Piper, Gibbs, and bivariate ionic relationships consistently indicate that the Ca–Mg–HCO₃ hydrochemical facies is dominant, representing recently recharged groundwater with short residence times. Silicate weathering of feldspars and ferromagnesian minerals is identified as the principal mechanism contributing major ions, while cation-exchange and reverse ion-exchange processes further modify ionic proportions within the weathered regolith and fractured basement.
2. The predominance of bicarbonate alkalinity suggests equilibration with soil CO_2_ and silicate weathering under acidic to near-neutral conditions, which are characteristic of humid tropical basement terrains that lack carbonate lithologies. The observed carbonate speciation reconciles pH, alkalinity, Piper facies, and saturation indices, thereby supporting the conclusion that groundwater chemistry is controlled primarily by water–rock interaction within the weathered regolith and fractured bedrock.
3. The CAI values indicate dominant cation–anion exchange, with Ca^2+^ and Mg^2+^ in groundwater exchanged with Na^+^ and K^+^ in host rocks, alongside base-exchange reactions.
4. Multivariate statistical analyses further confirm that groundwater chemistry is primarily influenced by water–rock interactions, while elevated NO_3_ and PO_4_ concentrations observed in certain locations indicate localized anthropogenic impacts resulting from agricultural activities and on-site sanitation.
5. Geochemical modeling indicates that all examined mineral phases are undersaturated, suggesting that mineral dissolution predominantly governs the major ion composition in groundwater.
6. Environmental isotope analysis reveals that local rainfall is the primary source of groundwater recharge in the study area. The δ^18^O and δ^2^H values of groundwater from boreholes and hand-dug wells plot close to both the GMWL and regional LMWL, indicating a meteoric origin derived from recent precipitation. The d-excess values (8.7–12.2‰), clustering around the global average of 10‰, further suggest minimal evaporation prior to recharge. These results collectively imply that modern rainfall is the main contributor to groundwater replenishment, with limited influence from evaporative enrichment or secondary processes.
7. Evaluation of the working hypotheses confirmed that (H₁) groundwater isotopic composition aligns closely with the regional meteoric line, indicating rapid, direct recharge from rainfall; (H₂) silicate weathering predominates, with undersaturated carbonates and limited buffering; and (H₃) hand-dug wells show greater isotopic enrichment due to shallower depths and dry-season evaporation. These verified hypotheses strengthen the conceptual understanding of groundwater evolution in the crystalline basement aquifers of Oye-Ekiti and provide a baseline for isotope-based monitoring and sustainable groundwater management in the area.

This study establishes a robust geochemical and isotopic baseline for Oye-Ekiti, representing groundwater from shallow and intermediate-depth aquifers sampled during the dry season. Because detailed borehole completion logs were unavailable, depth and construction information were inferred from regional datasets and literature. Consequently, the dataset best reflects near-surface hydrogeochemical and isotopic conditions that dominate household and community water supply systems. Potential dry-season effects include slight enrichment of δ^18^O and δ^2^H values in hand-dug wells and elevated solute concentrations due to reduced dilution. Future work should adopt a stratified sampling design with verified construction datasets and include both wet and dry season campaigns to enhance temporal and depth-based comparability of groundwater processes in the crystalline basement aquifers of Oye-Ekiti. The limitations of this study primarily stem from the lack of seasonal sampling campaign due to paucity of funds, absence of a comprehensive set of trace elements (e.g., As, Cd, Ni, Cr, Hg, Pb, Zn, etc.), contaminants of emerge concern (e.g., micro/nano-plastics, Perfluoroalkyl and Polyfluoroalkyl Substances—PFAS, Polycyclic Aromatic Hydrocarbons—PAHs, etc.), and environmental isotopes (e.g., ^3^H, ^14^C, δ^15^N, δ^53^Cr, ^87^Sr/^86^Sr, δ^11^B, etc.). These would enhance our understanding of groundwater resources in the Oye-Ekiti area, and we strongly recommend that future research focuses on these areas. Additionally, future studies on groundwater pollution in this region could address the following: (i) application of Machine (ML) and Deep Learning (DL) techniques utilizing qualitative and quantitate data, (ii) calculation of geo-environmental indices (e.g., Water Quality Index-WQI, Heavy Metal Pollution Index-HPI, molar ratios, etc*.*) to check the suitability of water for various uses and to further evaluate hydrogeochemical processes, (iii) analytical mineralogical and petrological studies via various techniques (e.g., X-Ray Diffraction-XRD, Scanning Electron Microscope-SEM) to identify mineral phases contributing dissolved chemical elements in groundwater, (iv) forward and inverse (mass balance) geochemical modeling using multiple scenarios, (v) spatiotemporal monitoring of groundwater quality and quantity on a monthly basis, (vi) use of mathematical receptor models (e.g., Positive Matrix Factorization-PMF, UNMIX) to further investigate the origin of the solutes, (vii) integration of spatial statistics with Geographical Information Systems (GIS) to find out information about spatial patterns of major/trace elements, (viii) determination of Natural Background Levels (NBLs) and Threshold Values (TVs) of specific chemical elements in combination with epidemiological data from local medical centers to conduct a successful Human Health Risk Assessment (HHRA), (ix) vulnerability studies using various methods (e.g., DRASTIC, GOD, SINTACS, etc*.*), and (x) application of Multi-Criteria Decision Making (MCDM) models (e.g., Analytic Hierarchy Process-AHP, SWOT analysis, Driver-Pressure-State-Impact-Response (DPSIR) framework, Field Force Analysis-FFA) for integrated water resources management.

Finally, water quality research should prioritize SDGs (Goals 3, 6, and 11), with an emphasis on optimal groundwater management, effective sanitation through waste control, continuous monitoring, and ensuring access to clean water to promote sustainable urban development.

## Supplementary Information

Below is the link to the electronic supplementary material.

Supplementary Material 1

## Data Availability

All data generated or analyzed during this study are included in this manuscript.
