# Supplementary material for: Hydrochemistry and environmental isotope signatures (δ18O and δ2H) in the crystalline basement groundwater system of Oye-Ekiti, southwestern Nigeria
Source: Sci Rep. 2026 Apr 27;16:19418. doi: 10.1038/s41598-026-50252-4 (PMC13287813; doi:10.1038/s41598-026-50252-4)
Supplement: Supplementary file 1 — Supplementary Material 1 [file 41598_2026_50252_MOESM1_ESM.docx]

| **ID** | **pH_CLR** | **Eh_CLR** | **EC_CLR** | **TH_CLR** | **Ca_CLR** | **Mg_CLR** | **Na_CLR** | **K_CLR** | **HCO3_CLR** | **Cl_CLR** | **SO4_CLR** | **NO3_CLR** | **PO4_CLR** | **TDS_CLR** |
| --- | --- | --- | --- | --- | --- | --- | --- | --- | --- | --- | --- | --- | --- | --- |
| BH-01 | 0.02 | -0.51 | 0.4 | 1 | 0.66 | 0.91 | 0.42 | -0.6 | 0.69 | -1 | -1.11 | -0.81 | 0.24 | 0.39 |
| BH-02 | 0.09 | -2.52 | 0.5 | -0.08 | 0.43 | -0.25 | 0.45 | -0.6 | 0.79 | -1.29 | -0.26 | 0.02 | -0.8 | 0.5 |
| BH-03 | 0.21 | 0.06 | -0.3 | 0.26 | -0.13 | 0.28 | 0.2 | 0.1 | -0.27 | -1.29 | -0.34 | -0.75 | -1.19 | -0.31 |
| BH-04 | -0.07 | -0.13 | -0.59 | -1.1 | -0.95 | -1.17 | 0.53 | -0.31 | -0.04 | -0.08 | -0.97 | -1.62 | 0.06 | -0.6 |
| BH-05 | -0.08 | 0.3 | -1.19 | -1.05 | -0.33 | -1.3 | -0.28 | -1 | -0.28 | -0.08 | -0.96 | -0.48 | -0.28 | -1.21 |
| BH-06 | -0.04 | 0.21 | 0.6 | 1.07 | -0.01 | 1.14 | 0.65 | -0.31 | 0.22 | 0.51 | -0.06 | 1.68 | -0.09 | 0.6 |
| BH-07 | 0.15 | -3.13 | 0.56 | 1.22 | 0.3 | 1.27 | 0.34 | -0.31 | 0.88 | -1.29 | -0.85 | 0.31 | 0.12 | 0.56 |
| W-01 | 0.07 | -0.08 | 0.32 | 0.89 | 0.47 | 0.82 | 0.62 | -0.6 | 0.46 | -0.59 | 0.82 | -0.27 | 0.13 | 0.32 |
| W-02 | 0.09 | -0.44 | 0.39 | 0.91 | -0.6 | 0.91 | 0.74 | -0.6 | 0.63 | 0.51 | -0.94 | -0.21 | 0.09 | 0.4 |
| W-03 | -0.05 | -0.09 | -0.7 | -1.49 | -0.7 | -0.06 | 0.2 | 0.1 | 0.12 | 0.01 | 0.39 | -1.47 | 0.08 | -0.7 |
| W-04 | 0.06 | 0.27 | -0.13 | 0.53 | -0.07 | 0.57 | -0.01 | -0.31 | 0.19 | -1 | -0.93 | -0.06 | 0.15 | -0.12 |
| W-05 | -0.04 | 0.24 | -0.67 | -0.64 | -0.07 | -0.84 | -0.65 | 0.38 | 0.15 | 0.01 | -0.41 | -0.02 | 0.12 | -0.67 |
| W-06 | 0 | 0.32 | -0.63 | -0.98 | -0.41 | -1.17 | -0.28 | 0.25 | 0.22 | -0.44 | -0.35 | -1.29 | 0.03 | -0.63 |
| W-07 | -0.02 | 0.37 | -0.86 | -0.16 | 0.66 | 0.44 | -1.46 | -0.09 | -0.23 | 0.1 | 1.26 | -0.48 | 0 | -0.86 |
| W-08 | -0.06 | 0.39 | 0.12 | 0.2 | -0.82 | 0.18 | 0.92 | 0.94 | -0.86 | 0.45 | 1 | 0.53 | 0.24 | 0.12 |
| W-09 | -0.06 | 0.41 | 1.33 | 1.43 | -0.01 | 1.43 | 1.49 | 1.35 | 0.29 | 1.57 | 1.41 | -2.31 | -0.02 | 1.33 |
| W-10 | -0.05 | 0.4 | 0.51 | 0.37 | 0.54 | 0.25 | 1.03 | 0.1 | 1.13 | 0.39 | -0.02 | 2.04 | 0.17 | 0.51 |
| W-11 | 0.02 | 0.38 | 0.99 | -1.32 | 0.19 | 0.22 | 0.34 | 0.7 | 0.19 | 1.02 | -0.11 | 1.8 | 0.05 | 0.99 |
| W-12 | 0.12 | 0.01 | 0.93 | 1.17 | 0.69 | 1.05 | 0.71 | -0.09 | 0.2 | 0.91 | -0.5 | 1.74 | 0.15 | 0.94 |
| W-13 | 0.01 | -0.38 | -0.01 | 0.02 | -0.7 | 0.06 | 0.53 | 0.79 | -0.17 | 0.41 | 1.06 | 0.74 | -0.42 | -0.01 |
| W-14 | -0.01 | -0.02 | 0.6 | 0.83 | 0.1 | 0.88 | 0.56 | 0.5 | -0.46 | 0.71 | -0.46 | -1.67 | 0.15 | 0.6 |
| W-15 | 0.05 | 0.03 | 0.28 | 0.59 | 0.38 | 0.57 | 0.68 | -0.6 | -0.13 | 0.26 | 0.86 | 0.43 | 0.19 | 0.28 |
| W-16 | -0.03 | 0.06 | -0.86 | -0.71 | -0.33 | -0.84 | -0.2 | 0.25 | -0.28 | -0.19 | -0.6 | 2.22 | 0 | -0.86 |
| W-17 | -0.01 | 0.16 | 0.62 | 0.76 | 0.59 | 0.71 | 0.1 | -0.6 | 0.22 | 0.66 | -0.17 | 0.02 | 0.01 | 0.62 |
| W-18 | -0.05 | 0.24 | -0.82 | -1.49 | 0.15 | -2.88 | -1.46 | -0.6 | -0.68 | -0.19 | -0.52 | -0.75 | 0.06 | -0.83 |
| W-19 | -0.04 | 0.19 | -0.67 | -1.95 | 0.4 | -2.76 | 0.05 | -1.7 | -0.34 | -0.19 | -0.15 | 0.35 | 1.18 | -0.67 |
| W-20 | -0.04 | 0.28 | -0.53 | 0.14 | 0.49 | 0.03 | -1.74 | -0.6 | -0.46 | 0.01 | -0.4 | -0.1 | 0.24 | -0.52 |
| W-21 | -0.07 | 0.3 | -0.62 | -0.34 | -0.82 | -0.32 | -0.2 | -1 | -0.28 | 0.01 | -0.96 | -0.11 | 0.37 | -0.6 |
| W-23 | -0.01 | -0.1 | -0.47 | -2.15 | -1.29 | -0.75 | -0.54 | 0.1 | -0.23 | -0.31 | -0.34 | -1.64 | 0.17 | -0.48 |
| W-24 | 0.06 | 0.03 | 0.48 | 0.58 | 0.61 | 0.36 | 0.45 | 0.94 | 0.46 | -0.44 | -0.18 | 0.56 | 0.1 | 0.47 |
| W-25 | 0.05 | 0.13 | -0.5 | 0.23 | 0.47 | 0.14 | -2.84 | 0.79 | -0.09 | -0.44 | -0.11 | 0.15 | 0.15 | -0.5 |
| W-26 | -0.02 | 0.14 | -0.25 | 0.36 | 0.36 | 0.31 | -1.05 | -0.31 | 0.04 | -0.19 | -0.55 | -0.52 | 0.09 | -0.26 |
| W-27 | -0.08 | 0.18 | -0.15 | 0.03 | -0.33 | 0.03 | 0.29 | 0.61 | 0 | 0.45 | -1.16 | 1.25 | 0.16 | -0.15 |
| W-28 | -0.01 | 0.29 | -0.35 | 0 | -0.7 | 0.04 | 0.15 | -0.31 | -0.34 | -0.44 | -0.36 | 0.28 | 0.1 | -0.36 |
| W-29 | 0.02 | 0.16 | 0.3 | -0.01 | 0.1 | -0.05 | 0.53 | 0.7 | 0.15 | -0.08 | 1.36 | 1.43 | 0.13 | 0.3 |
| W-30 | -0.13 | 0.31 | 0.87 | 0.86 | -0.26 | 0.93 | 1.11 | 0.38 | -0.96 | 0.95 | 1.36 | 1.8 | -0.22 | 0.87 |
| W-31 | -0.02 | 0.26 | 0.08 | 0.43 | 0.5 | 0.38 | 0.1 | -0.09 | 0 | -0.08 | 1.2 | -0.9 | 0.1 | 0.08 |
| W-32 | 0.05 | 0.25 | 0.42 | 1.2 | -0.5 | 1.29 | -0.36 | 0.1 | 0.52 | -0.44 | 0.13 | -1.69 | 0.24 | 0.42 |
| W-33 | -0.01 | 0.26 | 0.56 | -0.78 | 0.05 | -1.08 | 0.85 | -0.6 | 0 | 0.61 | -0.74 | 1.35 | -0.02 | 0.56 |
| W-34 | 0.06 | 0.34 | 0.72 | 0.42 | 0.1 | 0.99 | -0.28 | 0.61 | 0.1 | 0.61 | 0.78 | 0.84 | -0.05 | 0.72 |
| W-35 | -0.07 | 0.22 | 0.32 | -0.69 | 0.15 | -1 | 0.56 | 0.25 | -0.46 | 0.45 | 1.34 | 1.88 | -0.39 | 0.32 |
| W-36 | 0.01 | 0.18 | 0.05 | 0.18 | 0.41 | -0.09 | 0.34 | 0.61 | -0.31 | 0.2 | -0.12 | -2.18 | -1.09 | 0.04 |
| W-37 | -0.05 | 0.09 | -0.2 | 0.06 | 0.36 | -0.05 | -0.45 | -0.31 | -0.23 | 0.26 | -0.16 | 1.19 | 0.1 | -0.2 |
| W-38 | -0.05 | 0.09 | -0.65 | -0.24 | -0.37 | 0.16 | -0.2 | 0.25 | -0.53 | 0.17 | 0.9 | -0.9 | 0.15 | -0.65 |
| W-39 | -0.02 | 0.15 | -0.29 | -1.07 | 0.05 | -1.58 | -0.76 | 0.1 | -0.23 | -0.78 | 0.07 | 0.22 | -0.2 | -0.29 |
| W-40 | -0.05 | 0.23 | -0.27 | 0.31 | 0.4 | 0.25 | 0.05 | 0.61 | -0.04 | 0.18 | 1.16 | -0.99 | 0.09 | -0.26 |
| W-41 | -0.03 | 0.22 | -0.1 | 0.33 | 0.15 | 0.31 | 0.05 | 0.5 | -0.46 | 0.26 | 0.66 | 0.5 | -0.02 | -0.1 |
| W-42 | 0.03 | 0.11 | 0.06 | 0.76 | 0.1 | 0.8 | -0.45 | -0.09 | 0.65 | -0.08 | -0.41 | -0.63 | -0.02 | 0.06 |
| W-43 | 0.05 | 0.13 | 0.02 | -1.12 | 0.19 | -1.83 | -0.07 | -0.6 | 0.22 | -0.59 | -0.72 | -1.34 | 0.13 | 0.02 |
| W-22 | 0.03 | -0.96 | -0.21 | 0.26 | -0.6 | 0.31 | -1.74 | 0.25 | -0.15 | -0.19 | 0.14 | -0.15 | -0.76 | -0.21 |
